# Ion Valency as a Molecular Switch for Salt‐Resistant Underwater Adhesion

**DOI:** 10.1002/adma.202508666

**Published:** 2025-08-05

**Authors:** Chang‐Sheng Wang, Jiaxing Zhang, Hu Zhang, Wojciech Raj, Nahid Hassanpour, Duy Anh Pham, Hui Guo, Xingxun Liu, Heng Chang, Alexandre A. Arnold, Isabelle Marcotte, Rongxin Su, Wei Qi, Xavier Banquy

**Affiliations:** ^1^ Faculty of Pharmacy Université de Montréal Montréal Québec H3T 1J4 Canada; ^2^ Chemical Engineering Research Center School of Chemical Engineering and Technology Tianjin University Tianjin 300072 P. R. China; ^3^ State Key Laboratory of Chemical Engineering and Low‐Carbon Technology Tianjin University Tianjin 300072 P. R. China; ^4^ Institute of Biomedical Engineering Faculty of Medicine Université de Montréal Montréal QC H3C 3J7 Canada; ^5^ Lab of Food Soft Matter Structure and Advanced Manufacturing, College of Food Science and Engineering Nanjing University of Finance and Economics Nanjing 210023 China; ^6^ School of Marine Science and Technology Tianjin University Tianjin 300072 China; ^7^ Department of Chemistry Université du Québec à Montréal Montréal Québec H3C 3P8 Canada; ^8^ Department of Chemistry Faculty of Arts and Science Université de Montréal Montréal QC H3C 3J7 Canada

**Keywords:** adhesion, bottlebrush polymer, cation‐π interaction, molecular switch, molecular dynamics simulation

## Abstract

Achieving underwater adhesion remains challenging due to the disruption of interfacial interactions by hydration layers and the ionic environment. This study shows how high adhesion in a saline environment can be achieved in adhesive peptide systems relying on π–π and cation‐π interactions using multivalent ions. Monovalent ions (K^+^) disrupt native peptide‐peptide interactions, drastically reducing adhesion strength. Conversely, multivalent ions (Mg^2+^ and Y^3+^) enable robust interfacial adhesion by forming stable π‐cation‐π networks, effectively compensating for disrupted native pairings. The adhesion enhancement by Y^3+^ is particularly pronounced, highlighting its unique capability for multidentate bridging. Molecular dynamics simulations and quantum mechanical analyses confirm that Y^3+^ ions stabilize extended interfacial interactions, enabling stronger stress dissipation during tensile deformation. Additionally, NMR spectroscopy supports these observations by demonstrating significant cation‐dependent perturbations of aromatic (Phe) and cationic (Lys) peptide residues. A thermodynamic model further elucidates the competitive binding dynamics underpinning adhesion modulation and capturing all experimental trends. This work provides detailed molecular insights into ion valency effects on cation‐π mediated underwater adhesion, guiding the development of bio‐inspired materials with tailored ionic responsiveness suitable for biomedical and technological applications in saline environments.

## Graphical abstract

Monovalent K^+^ ions weaken adhesion by forming K^+^‐π complexes with weak bridging capacity. In contrast, multivalent cations like Y^3+^ enhance adhesion by forming stable π‐cation‐π bridging networks, which are resistant to salt and capable of compensating for the loss of native π–π and NH_3_^+^‐π interactions. Through these contrasting effects, K^+^ and Y^3+^ function as molecular switches that regulate adhesion in cation‐π‐based adhesive systems.

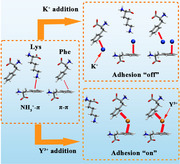

## 1 Introduction

Underwater adhesives hold great promise in biomedical applications such as tissue repair,^[^ ^1^ ^]^ wound closure,^[^ ^2^ ^]^ surgical procedures, and wearable medical devices.^[^ ^3^ ^]^ These applications often involve adhesion to hydrated surfaces or substrates exposed to biological fluids such as blood or tissue exudates. However, a critical challenge persists: the hydration layer that forms on wet surfaces disrupts interfacial interactions between adhesives and substrates, severely compromising adhesion strength. To address this limitation, researchers have turned to marine organisms such as mussels, barnacles, and sandcastle worms that have evolved sophisticated strategies to achieve robust underwater adhesion.

Among these natural models, mussels have emerged as a cornerstone for bioinspired adhesive design. Mussels secrete adhesive foot proteins rich in catechol‐functionalized amino acids, notably 3,4‐dihydroxyphenylalanine (DOPA).^[^ ^4^ ^]^ Catechol groups enable versatile binding through covalent crosslinking^[^ ^5^ ^]^ and non‐covalent interactions, including hydrogen bonding,^[^ ^6^ ^]^ electrostatic interactions,^[^ ^6a^ ^]^ metal coordination,^[^ ^7^ ^]^ π–π interactions,^[^ ^8^ ^]^ and cation‐π interactions.^[^ ^9^ ^]^ This multimodal adhesion mechanism allows mussels to overcome hydration barriers and adhere to diverse surfaces, from rocks to metallic substrates.^[^ ^10^ ^]^

Of these interactions, cation‐π bonding has garnered increasing attention. Cation‐π interactions occur between electron‐rich aromatic residues (e.g., phenylalanine, tyrosine, tryptophan) and positively charged ions or groups.^[^ ^11^ ^]^ These interactions are not only vital in biological processes such as protein‐ligand recognition but also offer a tunable design parameter for synthetic adhesives. Recent studies demonstrate that cation‐π‐mediated adhesion can be modulated by adjusting peptide composition, sequence, and ionic environments.^[^ ^11^, ^12^ ^]^ Cation affinity for aromatic groups follows the trend NH_4_^+^ ≈ K^+^ > Na^+^ > Li^+^, with potassium ions (K⁺) showing a pronounced impact on adhesion strength.^[^ ^13^ ^]^ Indeed, the vast majority of studies investigating the role of cation‐π interactions in adhesive systems have reported a dramatic decrease in adhesion strength with increasing ion concentration. This observation casts doubt on the significance of such interactions in marine environments, where salt concentrations are particularly elevated. Although significant progress in understanding the effects of monovalent cationic electrolytes on cation‐π adhesive systems has been made, the role of electrolytes valency remains poorly understood, despite its relevance to physiological fluids rich in multivalent ions (e.g., Ca^2+^, Mg^2+^, Fe^3+^, etc.). Multivalent ions are known to impact the polyelectrolyte structure via bridging effects, as well as the adhesive and lubrication properties.^[^ ^14^ ^]^ However, their potential to bridge π groups and enhance the adhesive properties of the peptide film remains unexplored. This knowledge gap limits the rational design of adhesives for real‐world biomedical scenarios, where ion valency and concentration dynamically influence interfacial interactions.

To address this gap, we used short peptides exhibiting strong cation‐π interactions in water and studied their interactions in the presence of electrolytes of different valencies. We used the surface forces apparatus (SFA) to measure the adhesive and cohesive forces between surface coated with the peptide of interest. Adhesive peptide (Ac‐GFKFKFGGGC‐amide) were first grafted via their cysteine residue to a poly(ethylene glycol) methyl ether methacrylate (PEGMA)‐based bottlebrush polymer (see the Supporting Information for detailed synthesis procedures and characterization data), and the resulting polymer was deposited on a mica surface prior testing in the SFA. The sequence of the peptide exhibits phenylalanine (F) and lysine (K) residues known to drive adhesion through K‐F (NH_3_^+^‐π) and F‐F (π–π or more precisely quadrupole‐quadrupole as highlighted in the work by Burley & Petsko^[^ ^15^ ^]^) interactions.^[^ ^11^, ^12^ ^]^ The use of a bottlebrush scaffold to deposit the peptide on the surfaces is motivated by the fact that these polymers have unmatched antiadhesive/antifouling properties, which make them the perfect substrate to study adhesive interactions between grafted moieties. By systematically varying the peptide grafting density on the bottlebrush polymer, ion valency (monovalent vs divalent vs trivalent), and ionic strength of the medium in which the surfaces are immersed, we evaluated the contributions of electrostatic screening and cation‐specific binding to adhesion. SFA measurements were rationalized using a mass‐balanced model linking ion concentrations to adhesion energy. Molecular dynamics (MD) simulations further revealed the mechanism of electrolyte valency modulating peptide‐peptide interactions by facilitating the formation of cationic bridges in the form of π‐cation‐π interactions. This work advances the fundamental understanding of cation‐π interactions in physiologically relevant ionic environments, providing actionable guidelines for optimizing adhesive performance in biological systems.

## 2 Results and Discussion

### 2.1 Peptide grafting on BB Polymer Modulates Side Chains Aggregation via Peptide‐Peptide Pairing

**Figure 1:**
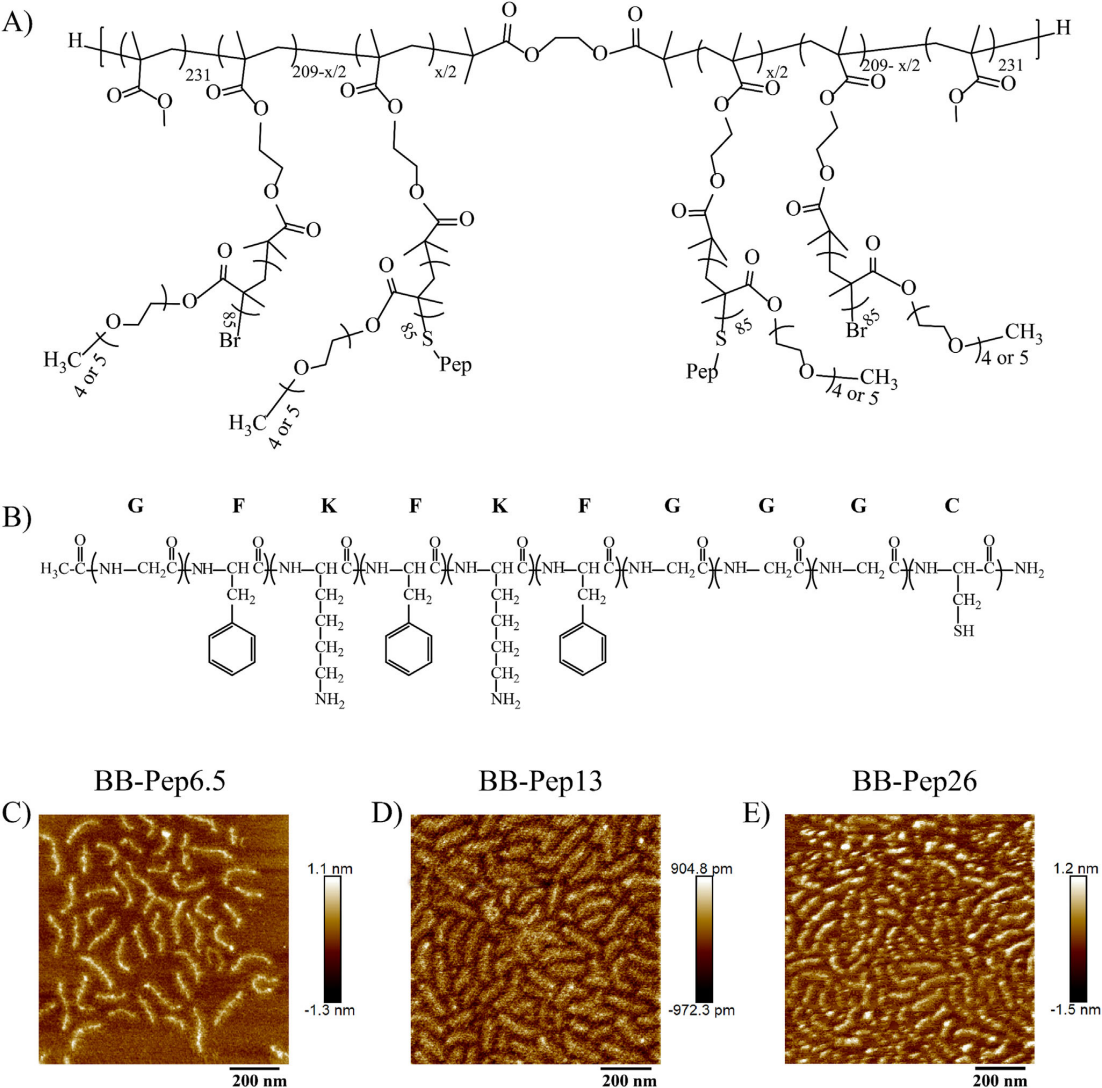
Chemical structure and polymer characterization: A) the chemical structure of the peptide sequence; B) the chemical structure of the synthesized BB‐Pep polymers and the morphology of the synthesized BB‐Pep polymers with respective grafting densities of C) 6.5%, D) 13% and E) 26%. The surfaces were prepared by drop casting a polymer solution at a concentration of 50 µg mL^−1^ followed by a gentle rinsing. The AFM images presented are the most representative ones.

The chosen peptide sequence, shown in **Figure** **1**A, composed of F and K amino acids, serves as a suitable model system for investigating cation‐π interactions.^[^ ^11^, ^12^ ^]^ The grafting procedure of the peptide at the termini of the BB polymer side chains was achieved by substituting the terminal bromide (Br) atom on the BB polymer with the thiol (─SH) group of the cysteine residue present in the peptide (Figure 1B). The peptide grafting density was precisely controlled by modulating the molar ratio of the peptide‐to‐bromide atoms. NMR spectroscopy confirmed actual grafting densities of 3.6%, 9.1%, and 24.3% (see Table S1 and Figures S2–S4 for details, Supporting Information).

The morphology of the resulting BB‐Pep conjugates deposited on a mica surface from a polymer solution at a concentration *C*_p_ = 50 µg mL^−1^ was characterized as a function of grafting density. The surfaces were uniformly covered by a monolayer of BB‐Pep, with surface coverage much higher at higher grafting densities (Figure 1C–E). This enhanced surface coverage correlates with improved adsorption affinity at higher peptide loading. Chain lengths remained consistent across all samples, as anticipated for polymers sharing identical backbones but differing in side‐chain peptide density.

At all grafting densities, the BB polymers exhibited a characteristic worm‐like shape with an extended backbone. This conformation (similar to a semi‐rigid stick) is likely due to the high side chain grafting density (50%) and the extended side chain length (DP = 85). Interestingly, at a peptide grafting density of 26%, distinct particle‐like structures emerged along the BB polymer (Figure 1E). This morphological change is attributed to intramolecular interactions, which is probably induced by the self‐assembly of grafted peptides driven by π–π and NH_3_^+^‐π interactions. The self‐assembly behavior was also commonly observed for other peptides,^[^ ^11^, ^12^, ^16^ ^]^ and can be used to engineer functional materials (e.g., self‐healing hydrogels).^[^ ^16^ ^]^

### 2.2 Peptide Grafting Density Modulates Adhesive and Cohesive Failure Mechanism

**Figure 2:**
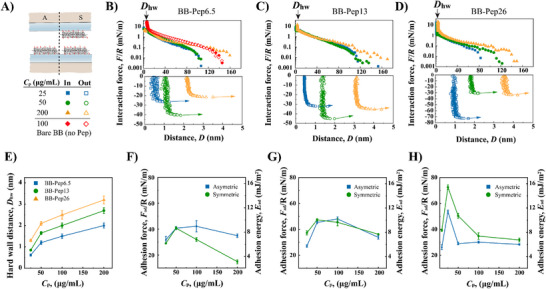
Interaction forces between BB‐Pep films under asymmetric and symmetric configurations. A) Schematic of asymmetric (one surface functionalized) and symmetric (both surfaces functionalized) configurations. Red represents the conjugated peptide. B–D) Force‐distance curves for symmetric configurations at varying polymer *C*_p_ for (B) BB‐Pep6.5 (low grafting density), C) BB‐Pep13 (medium grafting density), and D) BB‐Pep26 (high grafting density). E) Hard‐wall distance as a function of *C*_p_. F–H) Adhesion energy versus *C*_p_ for symmetric and asymmetric configurations: F) BB‐Pep6.5, G) BB‐Pep13, and H) BB‐Pep26. Data are shown as mean values ± SD (Standard Deviation) (*n* ≥ 6).

The interaction forces of BB‐Pep‐coated surfaces were systematically probed in asymmetric (BB‐Pep/mica) and symmetric (BB‐Pep/Pep‐BB) configurations (**Figure** **2**A). Adhesion, defined as the interaction between BB‐Pep and mica in asymmetric systems, arises from non‐specific electrostatic interactions between peptides and mica and depend on surface coverage and polymer conformation. Cohesion, measured between two BB‐Pep surfaces in symmetric systems, is driven by specific inter‐plane π–π and NH_3_^+^‐π interactions at the BB‐Pep/Pep‐BB interface.

Non‐conjugated BB polymers lacking adhesive peptides exhibit negligible adhesion (Figure 2B, red symbols) or are easily squeezed‐out upon compression, leading to direct mica‐mica contact. Grafting of the adhesive peptides onto the BB polymers enabled strong adsorption of the polymer to the mica surface, leaving non‐adsorbed peptides available to interact across the medium (Figure 2B–D).

In both symmetric and asymmetric configurations, the measured adhesive forces first increased with the polymer concentration *C*_p_ of the solution used to cover the surfaces by adsorption due to an increase in peptide availability. Beyond a critical *C*_p_, however, in‐plane steric interactions between adsorbed chains led to partially adsorbed BB polymer chains (and formation of loops/tails), increasing surface heterogeneity^[^ ^17^ ^]^ and reducing adhesion (Figure 2F–H). This conformational transition is evidenced in the force profiles by an increase of the onset of the repulsion force (Figure 2B–D) measured during compression and an increase in hard‐wall thickness (Figure 2E). The transition between these two adhesive regimes occurred at *C*_p_ values, which seem to be dependent on the peptide grafting density on the BB.

For the low grafting density polymer BB‐Pep6.5 (6.5% of the side chains carry a peptide moiety), adhesion (measured in the asymmetric configuration) reaches a maximum at *C*_p_ = 100 µg mL^−1^, an indication of surface saturation. In contrast, cohesion (measured in the symmetric configuration) peaks at a lower *C*_p_ (*C*_p_ = 50 µg mL^−1^) but remains weaker than the adhesion force measured in the asymmetric configuration. These observations are likely due to steric hindrance from non‐adhesive polymer chains and limited peptide density, favoring repulsive interactions over specific inter‐plane pairing. In other terms, in the asymmetric configuration, the peptides that are not adsorbed on the mica substrate can reach the facing surface and form a bridge, while in the symmetric configuration, this scenario is unfavored due to the presence of BB polymer on both surfaces. The low amount of peptide on the polymer does not favor pairing between peptides from facing surfaces either.

At higher peptide grafting densities, such as in BB‐Pep26, the system exhibits a distinct shift in behavior. Adhesion peaks at a lower concentration (*C*_p_ = 25 µg mL^−1^) in both configurations, suggesting earlier surface saturation at higher grafting density. In contrast to BB‐Pep6.5, cohesion in BB‐Pep26 exceeds adhesion, driven by a delicate balance between inter‐plane and in‐plane peptide pairing that emerges at high grafting densities. AFM imaging revealed that BB‐Pep26 chains display pronounced signs of intramolecular aggregation, facilitated by neighboring peptide‐peptide interactions. This in‐plane peptide pairing reduces the peptides' ability to adsorb onto the opposing mica surface but does not hinder their interaction with peptides from the facing surface. Consequently, in this scenario, cohesive forces measured in the symmetric configuration surpass those observed in the asymmetric case.

BB‐Pep13 exhibits intermediate behavior, where adhesion in both configurations is comparable. This further reinforces the idea of a gradual transition from non‐specific adhesion, driven by peptide adsorption onto mica at low grafting densities, specific inter‐plane cohesion, governed by peptide‐peptide pairing at higher grafting densities.

The peak cohesion of BB‐Pep films ranges from ≈8–16 mJ m^−2^, consistent with the cohesion of a peptide film derived from the bare FKFKF sequence, measured at 12.3 ± 3.4 mJ m^−2^.^[^ ^12b^ ^]^ Notably, this adhesion is achieved with significantly lower peptide amounts, highlighting the role of BB as a scaffold in enhancing peptide‐peptide pairing efficiency and enabling tunable adhesion/cohesion properties. To dissect the role of cation‐π interactions in BB‐Pep film cohesion, we grafted a control peptide, Ac‐GLKLKLGGGC‐amide, onto BB, replacing phenylalanine (Phe) with leucine (Leu) to eliminate aromatic π‐interacting groups. Under identical conditions (e.g., BB‐Pep13 at 50 µg mL^−1^), the control film exhibited an adhesion energy of 4.8 ± 0.3 mJ m^−2^, significantly lower than the 10.5 ± 0.5 mJ m^−2^ observed for the adhesive peptide Ac‐GFKFKFGGGC‐amide (Figure S8, Supporting Information). This substantial reduction underscores the critical contribution of cation‐π interactions to peptide‐mediated cohesion.

Our previous study^[^ ^12b^ ^]^ reported similar findings with a longer peptide sequence, FKFKFKFK, compared to a mutant sequence, LKLKLKLK, with Phe replaced by Leu. The FKFKFKFK film displayed strong adhesion (14.5 ± 4.2 mJ m^−2^), while the mutant showed significantly reduced adhesion (2.0 ± 0.7 mJ m^−2^).

Control peptides such as KKKKKKKK and FFFFFFFF, though not directly tested, can be inferred to exhibit weak cohesion based on related work.^[^ ^12^ ^]^ The KKKKKKKK sequence, composed solely of lysine (K) residues, is expected to have even lower cohesion than LKLKLKLK due to enhanced electrostatic repulsion among the positively charged K residues. Similarly, the FFFFFFFF sequence, relying solely on π–π interactions,s is anticipated to show weaker cohesion compared to hybrid sequences such as KKKKFFFF and FFKKFFKK, which incorporate both π–π and cation‐π interactions, exhibited low adhesion energies of 4.2 ± 0.1 mJ m^−2^ and 0.5 ± 0.4 mJ m^−2^, respectively,^[^ ^12^ ^]^ because cation‐π interactions are known to be energetically stronger than π–π interactions. These values remain far below the 14.5 ± 4.2 mJ m^−2^ observed for alternating FKFKFKFK films, consistent with the established energetic superiority of cation‐π interactions over π–π interactions.^[^ ^11^, ^18^ ^]^ These results, alongside the current data, provide robust evidence that cation‐π interactions are essential for the adhesive performance of peptide‐functionalized films, especially in systems designed with alternating cationic and aromatic motifs.

### 2.3 Effect of Contact Time and Aging on Adhesion

**Figure 3:**
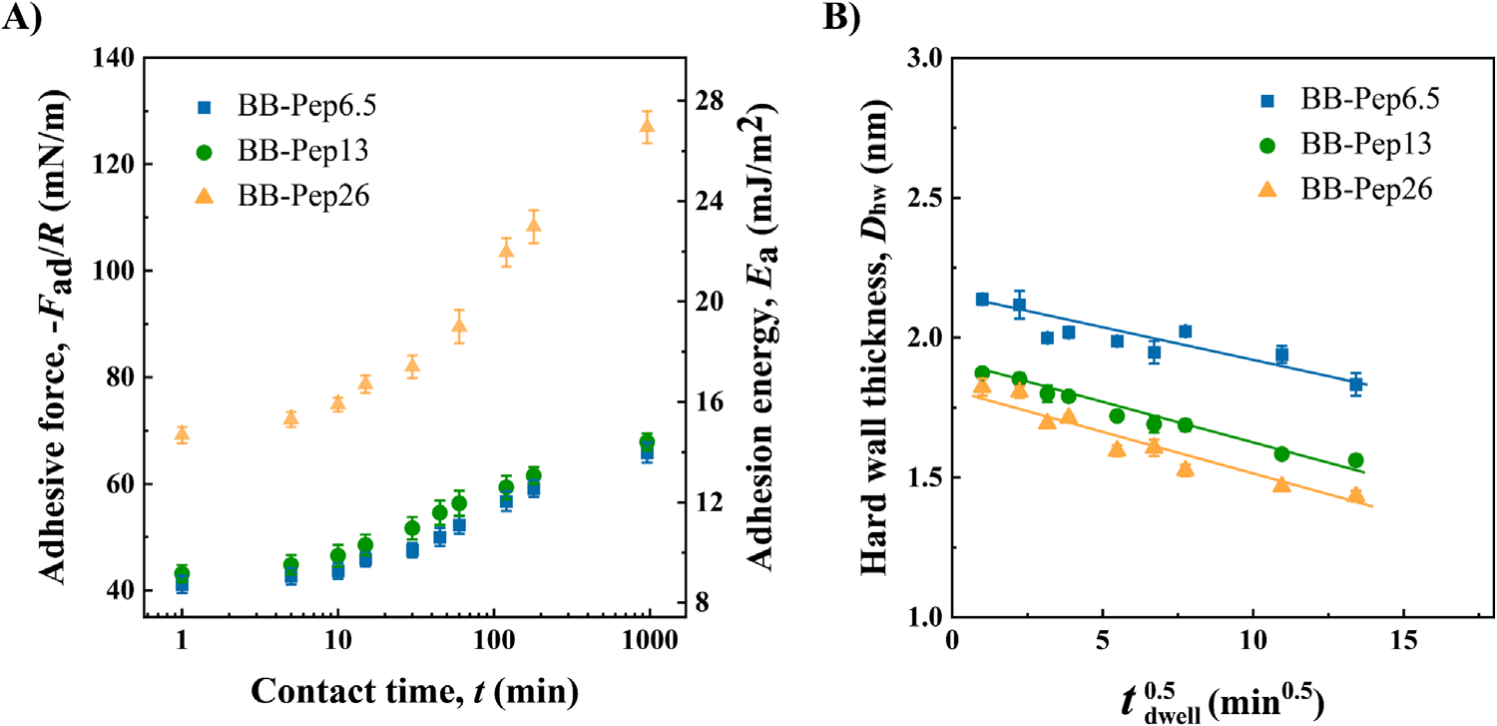
Time‐dependent adhesion and stability of BB‐Pep films. A) Adhesion energy (measured in symmetric configuration) as a function of time. B) Evolution of hard‐wall distance over time. Data are shown as mean values ± SD (Standard Deviation) (*n* ≥ 3).

To better understand the adhesion mechanism, we examined how contact time influences adhesion forces in symmetric configurations using surfaces prepared at optimal adhesion concentrations (*C*_p_ = 25–100 µg mL^−1^, depending on grafting density), where surface saturation is reached. As shown in **Figure** **3**A, adhesion forces in the symmetric configuration did not change during a short induction period (≈first 5 min), and rapidly rose over 2 h, suggesting progressive strengthening of intermolecular interactions (mediated by peptide pairing, brush interpenetration, and chain entanglement). Beyond 2 h, adhesion plateaued (Figure 3A), indicating equilibrium at a critical contact duration. As the adhesion forces increase with contact time, the adsorbed polymer layer thickness decreases linearly with the square root of time (Figure 3B), consistent with a diffusion‐limited process. This correlation supports the idea of gradual polymer layer consolidation, driven by slow chain diffusion and conformational relaxation. Notably, BB‐Pep26 (high peptide grafting density) exhibited a steeper rise of adhesion compared to lower grafting density polymers (BB‐Pep6.5&13; Figure 3A), suggesting that the peptide grafting density controls peptide‐peptide pairing when the surface is fully saturated. In addition, BB‐Pep samples showed excellent storage stability, with consistent adhesion and polymer size across various conditions (Figure S6, Supporting Information).

### 2.4 Impact of Ionic Strength and Cation Valency on the Pairing of Peptide‐Baring Surfaces

To elucidate the effect of ionic species on the interactions between surfaces baring adhesive peptides, we systematically investigated how ionic strength and cation valency modulate the adhesion of BB‐Pep‐coated polymer films.

**Figure 4:**
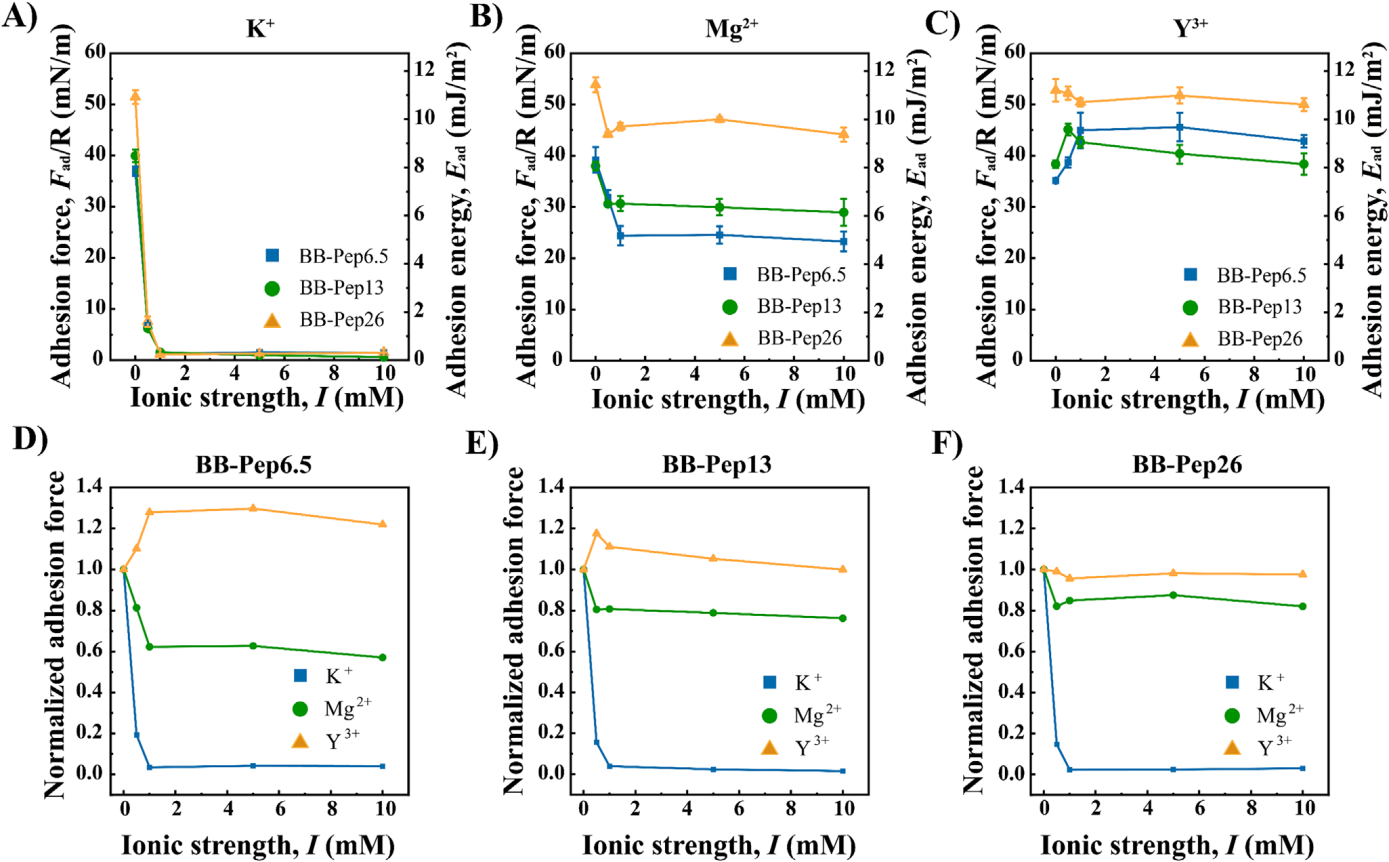
Impact of ionic strength and ion valency on the adhesion of BB‐Pep films. A–C) Adhesion energy between symmetric BB‐Pep‐coated surfaces (prepared at *C*_p_ = 50 µg mL^−1^) in different electrolyte solutions: A) monovalent (K^+^), B) divalent (Mg^2+^), and C) trivalent (Y^3+^) ions, tested for the three types of peptides baring BB polymers (BB‐Pep6.5, BB‐Pep13, BB‐Pep26). D–F) Dependence of adhesion on ion valency for D) low (BB‐Pep6.5), E) medium (BB‐Pep13), and F) high (BB‐Pep26) peptide grafting densities. Data are shown as mean values ± SD (Standard Deviation) (*n* ≥ 6).

As shown in **Figure** **4**A, monovalent K^+^ ions induce a strong loss of adhesion to near‐zero levels above 1 mm of ionic strength. This effect results from the competition between K^+^ ions in solution and lysine residues on the peptides, which disrupt the native π–π and NH_3_^+^‐π pairings between facing peptides. The pronounced sensitivity of NH_3_^+^‐π interactions to K^+^ aligns with prior studies.^[^ ^13^ ^]^ In contrast, exposure to divalent Mg^2+^ ions exhibits different behavior: at low ionic strengths (0.5–1.0 mm), adhesion decreases sharply at low ionic strengths (0–1.0 mm) and plateaus at higher ionic strengths (1.0–10 mm), as shown in Figure 4B. Trivalent Y^3+^ ions, however, uniquely enhance adhesion, inducing a local maximum (or an overshoot) at low ionic strengths (0.5–1.0 mm) for BB polymers with lower grafting densities (BB‐Pep6.5&BB‐Pep13; Figure 4C). Beyond 1 mm, adhesion gradually decreases.

To better describe the effects of ion valency on BB‐Pep at varying grafting densities, adhesion was normalized to the value at zero ionic strength for BB‐Pep at each grafting density (Figure 4D–F). For BB‐Pep6.5, Mg^2+^ addition reduces adhesion to ≈60% of its initial value, stabilizing between 1–10 mm (Figure 4D). Higher grafting density polymers (BB‐Pep13&26) retain up to 80% of their initial adhesion (Figure 4E–F). In contrast, Y^3+^ ions enhance adhesion by 20–25% at low ionic strengths (0.5–1.0 mm) and maintain 100–110% of the initial adhesion at high ionic strength (up to 10 mm), reflecting the robust bridging effects of multivalent ions. Monovalent ions primarily act as disruptors due to their low charge density, which limits bridging capacity.^[^ ^18^, ^19^ ^]^ The stronger π‐cation‐π bridges formed by multivalent ions can compensate for the loss of native interactions. Higher ion valency enhances bridging efficiency and strength due to increased charge density, which explains the adhesion overshoot observed for BB‐Pep6.5 and BB‐Pep13 with Y^3+^. However, at higher grafting density (e.g., BB‐Pep26), increased intra‐molecular peptide interactions create a thermodynamic barrier, requiring Y^3+^ ions to disrupt these pre‐existing complexes before forming inter‐surface bridges, thus reducing bridging efficacy and resulting in negligible adhesion overshoot.

Overall, the effects of ion valency stem from two competing processes: 1) disruption of native peptide pairing (e.g., π–π or NH_3_^+^‐π interactions) by cation‐π complexation and 2) formation of compensatory π‐cation‐π bridges. The low charge density of monovalent ions limits bridging capacity, thus behaving as adhesion disruptors. Multivalent ions, however, generate cation‐π complexes with sufficient charge density to enable robust intermolecular π‐cation‐π bindings, thereby preserving or even enhancing adhesion. On the other hand, peptide grafting density critically influences adhesive resilience. Higher densities (e.g., BB‐Pep26) enhance salt resistance by fostering interchain NH_3_^+^‐π and π–π interactions on individual surfaces. These intra‐plane interactions act as dynamic reservoirs for cation‐π complexes, which can redistribute to form inter‐plane π‐cation‐π bridges upon ion addition. This reservoir effect mitigates adhesion loss at higher ionic strengths, with its efficacy amplified at greater grafting densities. Consequently, systems with high peptide density exhibit a pronounced disparity in adhesion between monovalent and multivalent ions (Figure 4D–F), underscoring the synergistic role of intra‐surface interactions in stabilizing interfacial cohesion.

These findings also provide a mechanistic framework to rationalize why mussels evolutionarily favor multivalent metals like vanadium (V(III/IV)) and aluminum (Al^3+^) in their byssal adhesive systems.^[^ ^4^, ^20^ ^]^ High‐valency metals form strong, multidentate coordination bonds with catechol‐rich ligands (e.g., DOPA), resisting disruption in saline environments where abundant monovalent ions (e.g., Na^+^, K^+^) form weak, non‐bridging complexes. Divalent ions (e.g., Mg^2+^, Ca^2+^) offer intermediate bridging capacity but are less effective at high ionic strength, typical of seawater. Trivalent metals like vanadium and aluminum, through strong coordination complexes (e.g., octahedral tris–DOPA for V^[^ ^4c^ ^]^), achieve specific and resilient adhesion. Although cation‐π interactions do not involve defined coordination geometries like metal‐DOPA complexes, our findings suggest that multivalent ions (e.g., Y^3+^) can similarly stabilize π‐rich adhesive networks through bridging effects. This functional similarity highlights the evolutionary optimization of metal‐ligand and cation‐π interactions for robust adhesion in biological and synthetic contexts.

### 2.5 Evidence of the Modulation of Peptide‐Peptide Interactions by K^+^ and Y^3+^ From Multidimensional NMR Analysis

NMR spectroscopy provides detailed insights into molecular and intermolecular interactions of peptides assemblies in solution and enables a more comprehensive understanding of the roles of free electrolytes on intermolecular interactions.^[^ ^12^, ^21^ ^]^ Based on the composition of the peptide, the aromatic side chain of Phe (F) can participate in π–π stacking and cation‐π interactions with another peptide, while the protonated ε‐amino group (ε‐NH_3_^+^) of Lys (K) at physiological pH can engage in cation‐π interactions with Phe. The introduction of K^+^ (monovalent) and Y^3+^ (trivalent) cations provides a means to probe these interactions, as these ions may compete with Lys or alter the electronic environment of the aromatic system.

**Figure 5:**
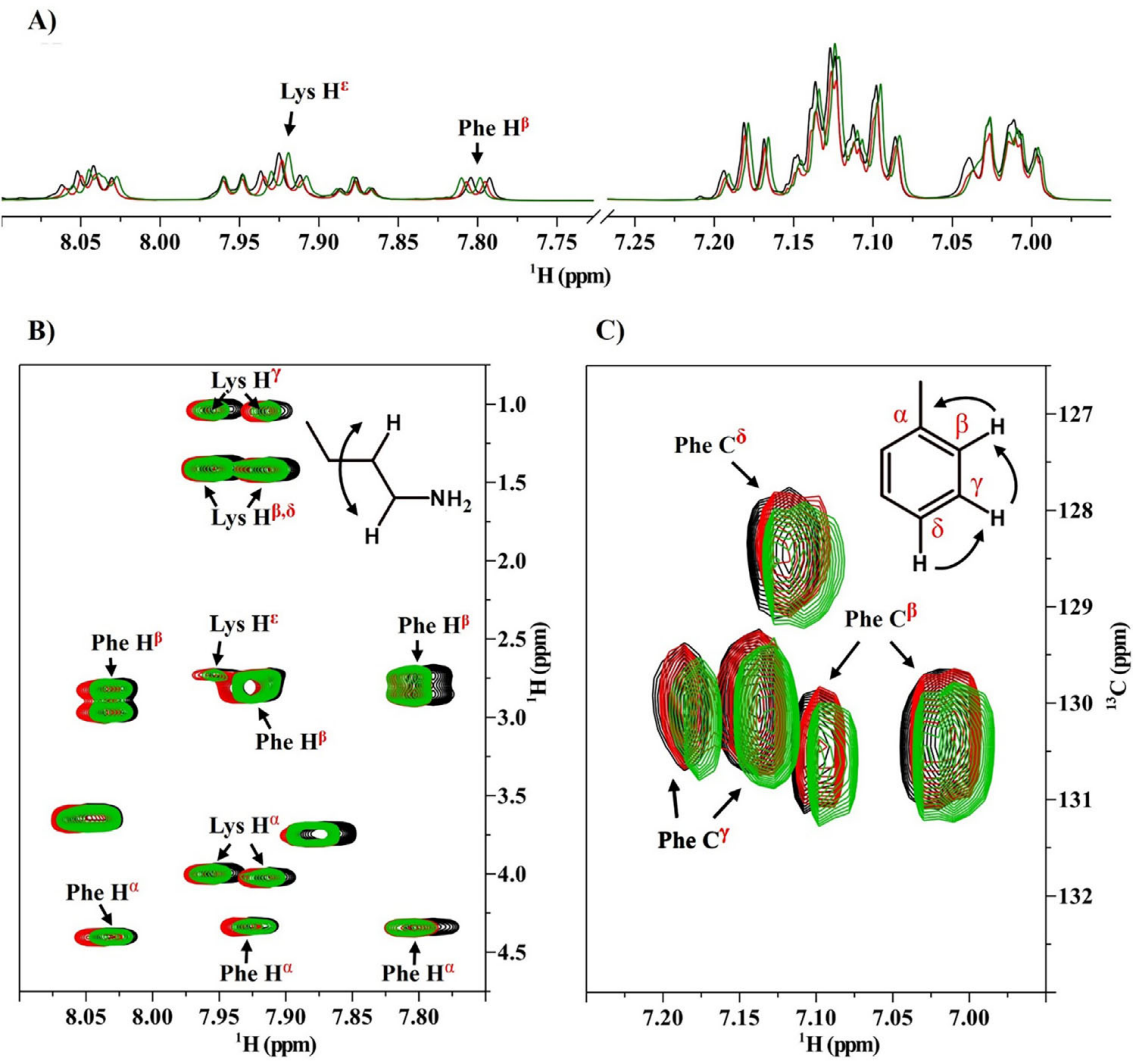
NMR evidence of π‐cation interaction between phenylalanine and lysine residues and its modulation in the presence of KCl and YCl_3_. A) The 1D ^1^H NMR spectrum for the peptide (5 mmol, black) in the presence of monovalent salt KCl (10 mmol, red) and trivalent salt YCl_3_ (10 mmol, green) in D_2_O:DMSO‐*d*_6_ (9:1 v/v; T = 300K). B) Expanded region of the 2D‐ ^1^H‐TOCSY spectrum showing correlations between lysine H^α^–H^ε^ protons and phenylalanine H^α^–H^β^ protons. C) Aromatic regions of the 2D‐^1^H, ^13^C‐HSQC spectrum displaying proton‐carbon ^1^J correlations. Arrows indicate chemical shift differences at the aromatic ring.

1D ^1^H NMR spectra revealed pronounced chemical shift perturbations in the aromatic region (7.00–7.30 ppm) and the amide region (7.70–8.10 ppm) upon addition of K^+^ and Y^3+^ (**Figure** **5**A; Figures S7–S9, and Table S2, Supporting Information). These shifts indicate the formation of cation‐π interactions between the introduced cations and the Phe aromatic rings. Notably, Y^3+^ induced more substantial perturbations compared to K^+^, consistent with its higher charge density, which enhances polarization of the π‐electron cloud. This observation underscores the high sensitivity of aromatic proton environments to cationic species valency.

Analysis of 2D‐^1^H,^1^H spectra (Figure 5B) demonstrates significant shifts in the Lys H^ε^ protons and Phe H^β^ protons in the presence of free cations. The perturbations of Lys H^ε^ suggest competitive displacement of the Lys's ‐NH_3_^+^ group from the Phe aromatic ring by added cations, disrupting pre‐existing Lys‐Phe cation‐π interactions. Concurrently, the Phe H^β^ shifts, proximal to the aromatic ring, reflect direct modulation of the ring's electron density upon cation binding. These data collectively indicate that both K^+^ and Y^3+^ interact with the Phe ring, with Y^3+^ exerting a stronger influence due to its trivalent charge.

^1^H‐^13^C heteronuclear couplings observed in 2D ^1^H‐^13^C HSQC (heteronuclear single quantum coherence) spectra (Figure 5C) corroborate alterations in the Phe aromatic system. Correlations between aromatic protons (H^δ^, H^β^, H^γ^) and carbons (C^δ^, C^β^, C^γ^) exhibited chemical shift deviations, particularly at C^β^ and C^γ^ positions, indicative of modified π‐electron density. The enhanced shifts with Y^3+^ align with its greater capacity to perturb the aromatic electronic environment. Furthermore, these perturbations imply potential disruption of π–π stacking between Phe residues, as the electronic complementarity required for such interactions is altered by cation binding.

Multidimensional NMR analyses provided evidence that K^+^ and Y^3+^ modulate both cation‐π and π–π interactions in peptide pairs. K^+^ partially disrupts Lys‐Phe cation‐π interactions and indirectly attenuates π–π stacking via electronic effects. In contrast, Y^3+^, with its elevated charge density, induces more pronounced displacement of Lys and significant electronic redistribution within the Phe ring, leading to greater disruption of both interaction types.

### 2.6 Thermodynamic Model of Adhesion in Presence of Free Electrolytes

Experimental data collectively show that the introduction of free cations in the medium shifts the interactions between peptides from π–π and cation‐π to peptide‐cation, depending on the valency of the ion. Multivalent ions promote the formation of π‐cation‐π bridges, thus preserving or even enhancing the adhesion between peptides. To support these experimental observations, we developed a thermodynamic mass‐balance model describing the adhesion force between peptide‐covered surfaces that accounts for the effects of both ionic strength and ion valency. The model considers that monovalent and multivalent ions influence the specific pairing process between peptides through the following equilibria:

(1) P+M↔PM

(2) P+T↔PT

(3) $$P+PT↔P_{2}T$$

(4) $$P+P↔P_{2}$$

where *P* represents the peptide, *M* the monovalent ion K^+^, and *T* the multivalent ion, specifically trivalent ion Y^3+^. The model rests on two critical assumptions: 1) all surface‐bound peptides are exclusively available for inter‐plane pairing, neglecting competitive intra‐plane interactions, this point will be discussed later in our analysis of the limitations of the model, and 2) adhesion arises solely from π–π, cation‐π and π‐cation‐π interactions, omitting contributions from secondary forces such as hydrogen bonding and van der Waals interactions. In the present model, adhesion is governed by the formation of peptide‐peptide pairing between the surfaces, and therefore by the concentrations of *P*_2_ and π‐cation‐π complexes *P*_2_ *T*.

To capture the interplay between ion valency and ionic strength, different apparent binding constants for each equilibrium reaction were defined as:

(5) $$K_{1}=\frac{\left[PM\right]}{\left[P\right]\left[M\right]}$$

(6) $$K_{2}=\frac{\left[PT\right]}{\left[P\right]\left[T\right]}$$

(7) $$K_{3}=\frac{\left[P_{2}T\right]}{\left[P\right]\left[PT\right]}$$

(8) $$K_{4}=K_{2}K_{3}=\frac{\left[P_{2}T\right]}{{\left[P\right]}^{2}\left[T\right]}$$

(9) $$K_{5}=\frac{\left[P_{2}\right]}{{\left[P\right]}^{2}}$$

Substituting Equation ((5), (6), (7), (8), (9)) into the conservation equation:

(10) $${\left[P\right]}_{T}=\left[P\right]+\left[PM\right]+\left[PT\right]+2\left[P_{2}T\right]+2\left[P_{2}\right]$$

where [*P*]_T_ is the total concentration of peptide adsorbed on the surfaces.

The final expression of the total concentration of species contributing to adhesion is:

(11) $$\left[P_{2}T\right]+\left[P_{2}\right]=\frac{\left(K_{4}\left[T\right]+K_{5}\right){\left[P\right]}_{T}^{2}}{{\left(K_{1}\left[M\right]+K_{2}\left[T\right]+1\right)}^{2}}$$

A detailed derivation of the governing equations is provided in the Supporting Information.

**Figure 6:**
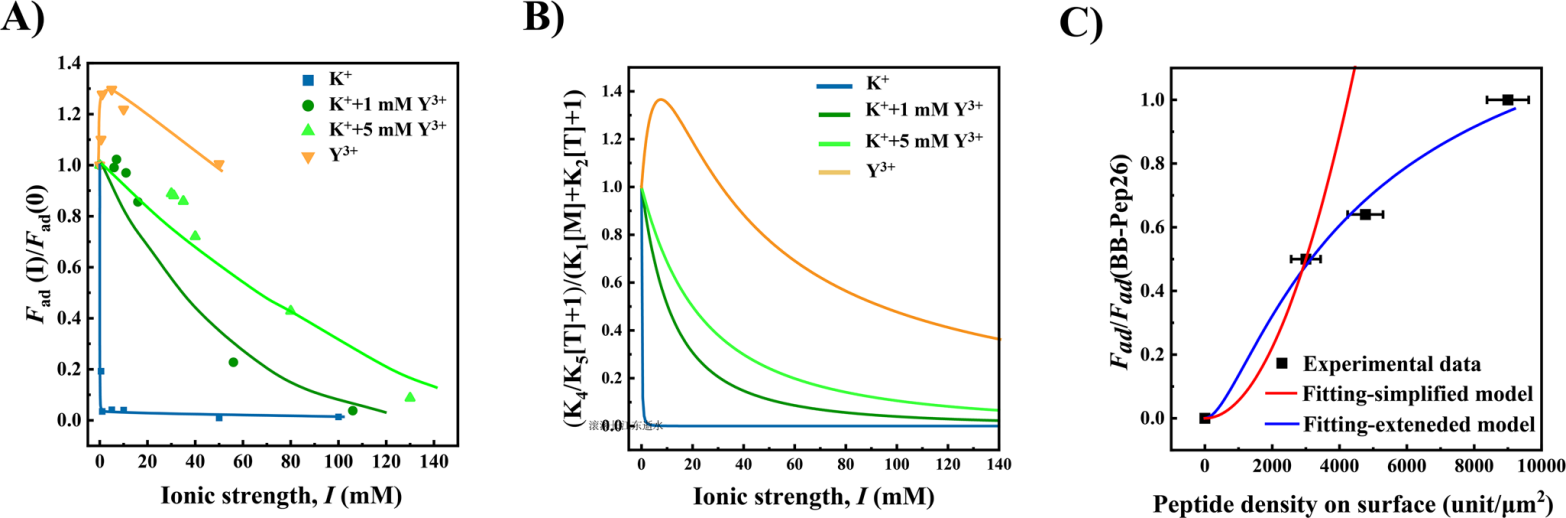
Comparison between experimental data and model fitting for BB‐Pep6.5. A) Adhesion force normalized by the adhesion force measured at *I* = 0 mm as a function of ionic strength for all cationic species and their mixtures; B) Theoretical predictions obtained from Equation 11 by using the fitted apparent equilibrium constants; C) Evolution of the adhesion force as a function of the peptide grafting density on the surface. The red curve represents a parabolic profile forced to pass through the experimental point at 3000 units µm^−2^, and the blue curve represents the fitting to the experimental data using an extended model that considers intra‐plane interactions. Peptide density on the surface was determined using AFM image analysis (based on ≥ 4 images) (Figure S10, Supporting Information) following the procedure described in Supporting Information.

Equation (7) can describe adhesion under varying conditions, accurately predicting trends as evidenced by the close agreement between experimental data (**Figure** **6**A) and theoretical values from the model (Figure 6B). However, deviations occur because the model does not account for intra‐plane pairings. These intra‐plane pairings can redistribute to form inter‐plane π‐cation‐π bridges upon ion addition, further complicating precise predictions. A detailed discussion is provided below.

#### 2.6.1 Effect of monovalent ions (K^+^) on adhesion

For monovalent ions ([*T*] = 0), Equation (11) simplifies to:

(12) $$\left[P_{2}T\right]+\left[P_{2}\right]=\frac{K_{5}{\left[P\right]}_{T}^{2}}{{\left(K_{1}\left[M\right]+1\right)}^{2}}$$

Equation (12) predicts an inverse square dependence of adhesion on monovalent ion concentration (*F*_ad_∼1/[*M*]^2^). This inverse square scaling underscores the extreme sensitivity of the adhesion to monovalent ion concentration, where even a slight increase in [*M*] induces a dramatic reduction in adhesive forces. Experimental data (Figure 6A, blue) align closely with Equation (8), as demonstrated by theoretical curves derived from the mass‐balance model (Figure 6B, blue). Such behavior is consistent with the proposed disruption mechanism, wherein K^+^ ions competitively bind to π electrons, forming low‐charge‐density cation‐π complexes that inefficiently bridge opposing surfaces. The rapid decline in adhesion reflects the dominance of this disruptive process over compensatory interactions, as monovalent ions lack the charge density required to stabilize robust π‐cation‐π networks. The fidelity of the model to experimental trends at lower ionic strengths further validates the key role of ion‐specific competitive binding in governing adhesive performance.

#### 2.6.2 Effect of trivalent ions (Y^3+^) on adhesion

For trivalent ions ([*M*] = 0), Equation (11) becomes:

(13) $$\left[P_{2}T\right]+\left[P_{2}\right]=\frac{\left(K_{4}\left[T\right]+K_{5}\right){\left[P\right]}_{T}^{2}}{{\left(K_{2}\left[T\right]+1\right)}^{2}}$$

Equation (13) effectively describes the influence of trivalent ions, capturing the characteristic adhesion overshoot observed at low ionic strengths. This is evidenced by the agreement between experimental data (Figure 6A, yellow) and theoretical predictions (Figure 6B, yellow). However, systematic deviations between experiments and theoretical curves emerge at elevated ionic strengths, where theoretical predictions underestimate experimental adhesion values. This discrepancy likely originates from two key factors not incorporated into the model's framework.

First, the model neglects the role of intra‐plane specific pairing interactions between adsorbed peptides on the same surface. These interactions act as reservoirs for cation‐π complexes upon ion addition, enabling a dynamic redistribution of complexes. As ionic strength increases, the transition of cation‐π complexes from intra‐plane to inter‐plane configurations amplifies the total population of π‐cation‐π complexes bridging opposing surfaces. This redistribution enhances experimental adhesion beyond model predictions, particularly for multivalent ions like Y^3+^. In contrast, monovalent K^+^ ions exhibit minor influence due to their inherently weak capacity to stabilize π‐cation‐π complexes, leaving the model's accuracy for K^+^ largely preserved. Second, the model assumes that adhesion is governed solely by cation‐π, π–π, and π‐cation‐π interactions, omitting contributions from secondary forces such as hydrogen bonding and van der Waals interactions. These non‐electrostatic interactions, which are relatively insensitive to ionic strength or ionic species, may provide additional adhesive reinforcement at higher ionic strengths.^[^ ^13^ ^]^ Their exclusion from the model could further explain the observed underestimation of adhesion in Y^3+^‐dominated systems.

#### 2.6.3 Mass‐balance model captures the evolution of adhesion in the presence of competitive binding

To further validate the model, we investigated the adhesion of BB‐Pep films in mixed electrolyte solutions containing both K^+^ and Y^3+^ (Figure 6A,B, green color). When the Y^3+^ concentration was maintained at 1 mm, adhesion exhibited a gradual decrease with increasing ionic strength. Nevertheless, this decay was significantly attenuated compared to the K^+^‐mediated system, particularly at low ionic conditions (Figure 6A, dark green). Elevating the Y^3+^ concentration to 5 mm further suppressed the decay, underscoring the enhanced stability of Y^3+^‐stabilized π‐cation‐π complexes under high‐salinity environments (Figure 6A, bright green). This behavior contrasts sharply with the K^+^ system, where adhesion diminished rapidly even at very low ionic strengths. The resilience of Y^3+^‐based adhesion highlights the critical role of multivalent cations in forming robust, salt‐resistant complexes through strong π‐cation‐π interactions. These findings demonstrate that increasing both cation valency and concentration reinforces inter‐plane interactions, mitigating ionic screening effects.

Equation (11) successfully reproduced the overall dependence of adhesion with electrolyte solution composition, capturing the transition from monovalent‐dominated disruption to trivalent‐stabilized reinforcement (Figure 6B, dark and bright green). However, consistent with its limitations in modeling pure Y^3+^ systems, the theoretical predictions systematically underestimated experimental adhesion values. This persistent discrepancy again suggests that unmodeled mechanisms, such as intra‐plane specific pairing interactions and their redistribution effects, contribute significantly to interfacial pairing.

#### 2.6.4 No electrolyte condition

In the case of no added electrolytes ([*P*_2_ *T*] = 0,  [*M*] = 0 and [*T*] = 0), Equation (16) becomes:

(14) $$\left[P_{2}\right]=K_{5}{\left[P\right]}_{T}^{2}$$

This expression predicts adhesion as a quadratic function of peptide grafting density, but it fails to capture the experimental trend where adhesion growth slows at higher surface coverage (Figure 6C). This discrepancy arises from neglecting intra‐plane pairing interactions, which become more significant at elevated grafting densities. Additionally, surface adsorption of peptides reduces the pool of peptides available for inter‐plane pairing, complicating the quantification of active adhesive groups. However, this deviation enables estimation of the fraction of peptides engaged in intra‐plane pairing and adsorption.

The red curve in Figure 6C, representing Equation (14), is adjusted to pass the experimental data at 0 and ≈3000 peptides µm^−2^, despite likely intra‐plane pairing at this grafting density. As surface coverage increases, Equation (14) increasingly overestimates the adhesion force due to increased intra‐molecular pairing and surface adsorption, which reduce the availability of peptides for inter‐plane bonding. By analyzing deviations between theoretical and experimental values, we estimate that ≈28% and 53% of peptides are engaged in intra‐plane pairing and adsorption at higher surface coverage. This increasing overestimation underscores the model's declining accuracy at high grafting densities and highlights the need to account for intra‐surface effects in predictive models.

To address these limitations of the initial quadratic model, we developed an extended model incorporating intra‐plane pairing interactions by introducing a new equilibrium:

(15) $$P+P↔P_{2,intra}$$

The corresponding apparent binding constant is defined as:

(16) $$K_{6}=\frac{\left[P_{2,intra}\right]}{{\left[P\right]}^{2}}$$

In the absence of ions, the conservation equation becomes:

(17) $${\left[P\right]}_{T}=\left[P\right]+2\left[P_{2}]+2[P_{2,intra}\right]$$

This leads to the expression:

(18) $$\left[P_{2}\right]=\frac{K_{5}{\left[P\right]}_{T}^{2}}{{\left(1+2K_{6}{\left[P\right]}_{T}\right)}^{2}}$$

(See Supporting Information for detailed derivation.)

Compared to Equation (14), Equation (18) has a denominator term that captures the competitive binding on crowded surfaces. As peptide grafting density increases, a larger fraction of peptides engages in intra‐plane pairing, reducing the pool available for inter‐plane adhesion. This term effectively moderates the quadratic dependence on surface coverage predicted by Equation (14), aligning theoretical predictions with the observed plateau in adhesion at higher densities (Figure 6C, blue line).

The improved fit of Equation (18) to experimental data highlights the significant role of intra‐plane interactions, particularly beyond ≈3000 peptides/µm^2^, where up to 53% of peptides are estimated to be engaged in intra‐plane pairing or surface adsorption. By incorporating this competition, the extended model accurately captures the non‐linear saturation observed in experimental data and provides a quantitative framework to estimate the fraction of peptides sequestered in intra‐plane interactions.

### 2.7 Structural Insights of Peptides Pairs by Molecular Dynamics And Quantum Mechanics

To gain deeper insights into how ion valency modulates cation‐π interactions, we performed MD simulations using a simplified model composed of surfaces functionalized with grafted polymer‐peptide chains at low peptide density. This simplified configuration was specifically designed to mimic the stress‐strain behavior of BB‐Pep6.5‐coated surfaces under tensile deformation in the presence of either K^+^ or Y^3+^ ions.

**Figure 7:**
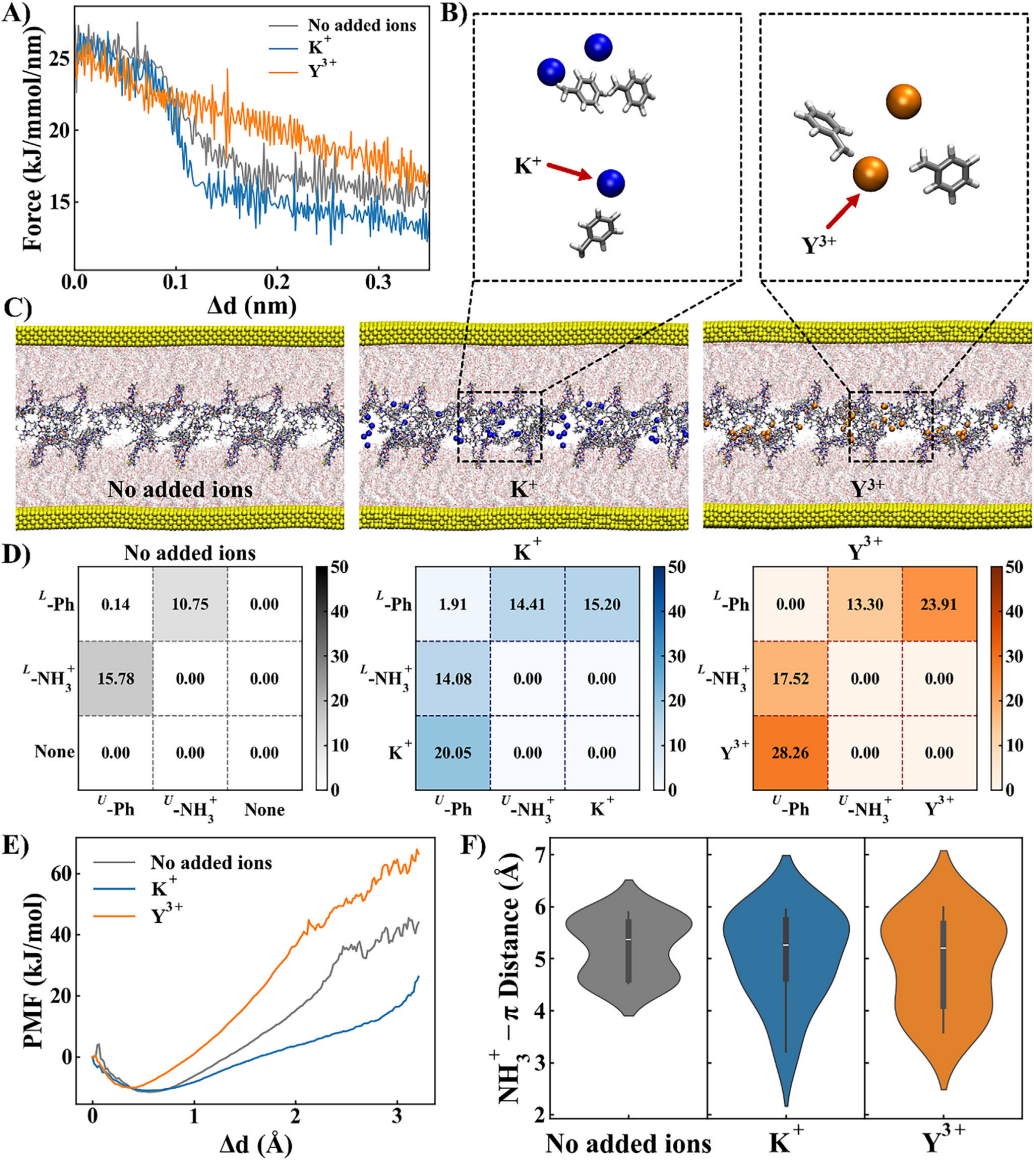
Pulling simulations of peptide‐bearing brushes under different conditions: no added ions, adding monovalent ions (K^+^) and trivalent ions (Y^3+^). A) Stress‐strain profiles during tensile separation, showing rapid force decay in K^+^‐containing simulations versus sustained dissipation with Y^3+^; B) Molecular‐level coordination of ions (K^+^ and Y^3+^) with phenylalanine (Ph) groups; C) Equilibrium conformations after pushing to contact, illustrating cation‐π structural arrangements. D) Quantified contact analysis (intra‐plane vs inter‐plane) between Ph groups in upper (^U^) and lower (^L^) polymer layers. E) Potential Mean Force (PMF) from umbrella sampling simulations on the three systems. (F) NH_3_^+^‐π distance distribution among the three systems.

The simulation setup involved two gold surfaces functionalized with polymer side chains, grafted at one end to the surface and at the other end with adhesive peptides at relevant grafting densities (Figure S11A–C, Supporting Information). The simulation consisted in pushing the surfaces into contact with subsequent equilibration and finally pulling the surfaces while monitoring the adhesive forces. Pulling dynamics revealed distinct mechanical responses depending on the ionic species present in the medium (**Figure** **7**A). K^+^‐containing systems exhibited rapid force decay within 0.1 nm of strain, indicative of weak interfacial bonding. In contrast, Y^3+^‐containing systems demonstrated sustained stress dissipation over extended strain intervals and higher peak stresses, reflecting robust interfacial interactions.

**Scheme 1:**
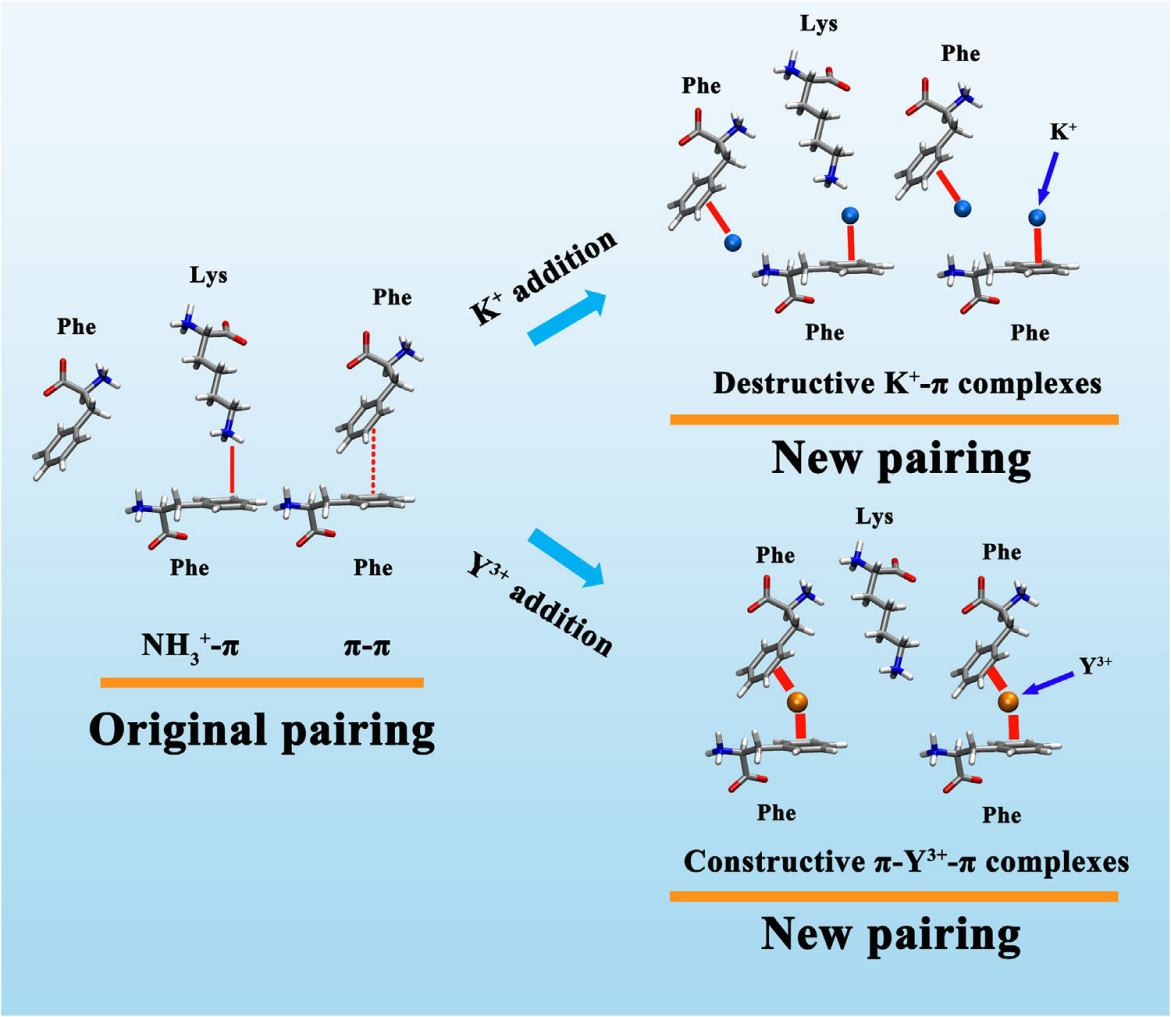
Diagram illustrating cation‐π (Lys‐Phe) and π–π (Phe‐Phe) interactions in BB‐Pep systems. In the absence of ions, native pairing involves cation‐π and π–π interactions. The addition of K^+^ or Y^3+^ replaces these with new cation‐π complexes due to their stronger interaction energies with Phe. K^+^ forms cation‐π complexes with limited bridging capacity, disrupting adhesion. Conversely, Y^3+^, leveraging its higher charge density, forms robust π‐cation‐π networks, preserving or enhancing adhesion in BB‐Pep films.

Detailed structural analyses (Figure 6B,C) revealed distinct polymer layers with grafted peptides forming inter‐layer contacts. K^+^ ions partially disrupted phenyl ring contacts, with the phenyl/K^+^ ratio of 1:1. This revealed that the addition of K^+^ ions replaced the π–π interactions with cation‐π interactions and thus decreased the attraction forces between BB‐peptide layers (**Scheme** **1**). In contrast, Y^3+^ coordinated multiple phenylalanine groups, forming stable π‐cation‐π architectures that reinforced interfacial connectivity. This multivalent bridging mechanism underlies the enhanced salt resistance and adhesion observed experimentally in Y^3+^‐added systems.

Free energy landscapes (FELs) of π–π interactions, quantified through center‐of‐geometry (COG) distances and dihedral angles between phenyl rings, provided insights on the geometry of the formed complexes (Figure S12, Supporting Information). In ion‐free systems, π–π pairs predominantly adopted T‐shaped geometries (dihedrals: 50°–90°). Addition of K^+^ broadened the dihedral distribution (0°–90°) and increased COG distances, reflecting partial disruption of π–π contacts. Y^3+^ induced intermediate effects, expanding dihedral angles to 25°–90° while maintaining COG distances near 7.5 Å (Figure S12, dashed box, Supporting Information), suggesting a balance between ion‐mediated disruption and compensatory bridging.

Interaction energy calculations quantified cation affinities for phenyl rings, with van der Waals strengths of −28.1 kJ mol^−1^ for NH_3_^+^), −54.2 kJ mol^−1^ for K^+^, and −61.2 kJ mol^−1^ for Y^3+^ (Figure S13, Supporting Information). These results align with NMR data, which show stronger Y^3+^‐induced chemical shift perturbations (Figure S9 and Table S2, Supporting Information), reflecting stronger cation‐π interactions. Quantitative contact analysis (Figure 7D) further confirmed Y^3+^’s superior binding to π electrons. For all the systems, cation‐π interactions predominate over π–π interactions. This is consistent with prior discussions that π–π interactions contribute minimally to cohesion.

To fully equilibrate the system during pulling simulation to consider the effects of interfacial rearrangement, umbrella sampling (US) simulations were performed. US studies revealed a higher potential mean force (PMF) for BB‐Pep6.5/Y^3+^ systems compared to BB‐Pep alone, while BB‐Pep6.5/K^+^ systems exhibited the lowest PMF (Figure 7E; Figure S14, Supporting Information). These simulations, incorporating equilibrated stress response and interfacial rearrangement, generated considerable PMF profiles that corroborate earlier findings on the influence of ion valency. Additionally, we also analyzed the distance distribution between ε‐NH_3_^+^ group of lysine and the benzene ring of phenylalanine in each system (Figure 7F). The introduction of K^+^ and Y^3+^ induced a broader distance distribution compared to the ion‐free system, characterized by shorter minimum and larger maximum distances. This broadening is due to the cation‐induced rearrangement of F and K residues. The observed changes reflect the disruption of native π–π interactions and NH_3_^+^‐π interactions and the formation of new cation‐π complexes.

## 3 Conclusion

The adhesion of BB‐Pep films is governed by peptide grafting density, ion valency, and ionic strength, which collectively modulate interfacial interactions. At low grafting density, asymmetric configurations yield higher adhesion due to favorable inter‐plane pairing, whereas symmetric configurations dominate at high grafting density, driven by enhanced out‐plane interactions. Monovalent ions (e.g., K^+^) disrupt adhesion by cation‐π complexes with weak bridging capacity that compete with native π–π and NH_3_⁺‐π interactions. In contrast, multivalent ions (e.g., Mg^2+^ and Y^3+^) enhance adhesion through robust π‐cation‐π networks, compensating for losses of native interactions. Intra‐plane pairing, while competing with inter‐plane bonding, stabilizes adhesion under ionic competition by enabling cation redistribution.

Integrated computational, experimental, and molecular dynamics analyses highlight the pivotal role of Y^3+^ multivalency in mitigating adhesion loss in high‐ionic‐strength environments. Its strong interaction energy with π groups and superior bridging capability ensure adhesion resilience. A thermodynamic model accurately captures experimental trends, but at high surface coverage (e.g., above ≈3000 peptides µm^−^^2^), intra‐plane interactions reduce available peptides for inter‐plane bonding. An extended model significantly improves predictions by accounting for this competition, aligning with the observed adhesion plateau. These insights advance the rational design of cation‐π driven adhesives, offering a robust framework for optimizing stability in physiological or marine environments where fluctuating ionic strength often compromises performance.

## 4 Experimental Section

### Materials

The adhesive peptide Ac‐GFKFKFGGGC‐amide (Pep) and control peptide Ac‐GLKLKLGGGC‐amide were synthesized at a 99% purity at the peptide synthesis core facility at Université de Sherbrooke. Salts (KNO_3_, Mg(NO_3_)_2_, Y(NO_3_)_3_; 99.99%, Sigma Aldrich) were used to probe monovalent (K^+^), divalent (Mg^2+^), and trivalent (Y^3+^) cation concentrations. Ultrapure water (18.2 MΩ·cm) and atomically flat muscovite mica (Grade 1, S & J Trading Inc.) served as solvent and substrate, respectively. All other reagents were from Sigma Aldrich Canada, unless otherwise stated.

### Atomic Force Microscopy

A 50 µg mL^−1^ polymer solution droplet was deposited onto a freshly cleaved mica substrate and allowed to adsorb for 1 h. Following adsorption, the substrate was rinsed extensively with Milli‐Q water to eliminate non‐adsorbed polymer. The morphology and surface coverage of the adsorbed polymer were then characterized using atomic force microscopy (AFM; Bruker Nanoscope V Dimension Icon/Fastscan) in PeakForce Tapping mode under ambient conditions. Measurements were conducted with silicon tips mounted on nitride cantilevers, featuring a resonance frequency of 50–90 kHz and a spring constant of ≈0.4 N m^−1^. The morphology of each sample was checked on 2–4 independent surfaces, with at least three areas examined on each surface. The AFM images presented are the most representative ones.

### Surface Force Measurement

Back‐silvered mica surfaces were affixed to cylindrical disks (2 cm curvature) using optical‐grade epoxy (Epon 1004F) under a laminar flow hood. The disks were mounted in a cross‐cylinder configuration within a multimodal miniature surface forces apparatus (µSFA, SurForce LLC, USA). A reference contact separation distance (*D* = 0) was established by bringing bare mica surfaces into adhesive contact in air. After separation, polymer‐coated surfaces were prepared as follows:

### Surface Force Measurement—Symmetric Surface Preparation

A BB‐Pep solution was injected between the mica surfaces and allowed to adsorb for 1 h. Surfaces were gently rinsed 4–5 times with Milli‐Q water to remove unbound polymer. A 50 µL droplet of Milli‐Q water was introduced between the surfaces for force measurements.

### Surface Force Measurement—Asymmetric Surface Preparation

The upper disk was replaced with an uncoated dummy disk stored in Milli‐Q water to prevent contamination. The symmetric coating protocol was repeated on the lower disk. The dummy disk was subsequently replaced with the original upper disk, and a 50 µL droplet of Milli‐Q water was injected for measurements.

A water bath within the SFA chamber maintained a saturated vapor environment to minimize evaporation during experiments. The separation distance (*D*) between surfaces was quantified via multiple‐beam interferometry by analyzing fringes of equal chromatic order (FECO) using a spectrometer. FECO wavelengths were converted to separation distances via a three‐layer interferometric model. Normal interaction forces (*F*) were recorded as a function of *D* during compression (approach) and separation (retract) cycles at a constant speed of 1 nm s^−1^. The adhesion or cohesion energy per unit area (*E*_a_ or *E*_c_) between two surfaces was defined by *F*_a_/*R* = 1.5π*E*_a_ or *F*_c_/*R* = 1.5π*E*_c_, where *R* is the surface radius of curvature of the surfaces. FECO fringes were processed using a custom MATLAB algorithm. All experiments were replicated ≥3 times at distinct contact positions across ≥2 independently prepared surfaces.

### Nuclear Magnetic Resonance

For Nuclear Magnetic Resonance (NMR) analysis, peptide samples were prepared in an H_2_O:D_2_O:DMSO‐*d*_6_ solution (72:18:10 by volume) at a concentration of 5 mg ml^−1^. The addition of 10% DMSO‐*d*_6_ was required to ensure the correct solubilization of the peptide. The monovalent (KCl) and trivalent (YCl_3_) salt solutions were prepared at concentrations of 1, 10, and 100 mm, while maintaining the peptide concentration at 5 mg mL^−1^.

All spectra were recorded at room temperature on a Bruker Avance III HD spectrometer operating at a field of 14.1 T with corresponding ^1^H and ^13^C Larmor frequencies of 599.99 and 150.87 MHz, respectively, using a double resonance BBFO probe. The high‐resolution ^1^H NMR spectra were obtained with water suppression by excitation sculpting, followed by 1.4 s acquisition time, a spectral width of 12 kHz, a recycle delay of 5 s, and 48 scans accumulated. The peptide resonances were assigned using the 1D ^1^H NMR spectra and 2D TOCSY and ROESY spectra obtained with mixing times of 150 and 250 ms, respectively. The TOCSY spectra were recorded with a spectral window of 8 kHz, a recycle delay of 2 s, 8 scans, and 256 increments in the indirect dimension. The ROESY spectra were recorded with spectral windows of 6 kHz, a recycle delay of 2 s, 16 scans, and 256 increments in the indirect dimension. All amino acids could be assigned, although no sequential assignment could be obtained due to the highly repetitive nature of the sequence. These ^1^H NMR spectra were also used to assess the effect of salt concentration on the peptides. To further monitor the effect of salt concentration on ^13^C nuclei, HSQC spectra were recorded with spectral windows of 6 (^1^H) and 27 kHz (^13^C), a recycle delay of 1.3 s, 16 scans, and 324 increments in the indirect dimension. All spectra were referenced using the internal DMSO residual signals at 2.5 and 39.5 ppm for the ^1^H and ^13^C NMR spectra, respectively. The spectra were analyzed using MNova14.3 software (Mestrelab Research, S.L.U., Santiago de Compostela, Spain).

### Molecular Dynamics and Quantum Mechanics Simulations—Simulation Setup for BB‐Pep Interactions

The BB‐Pep was modeled as a simplified system as polymer sidechains directly grafted to the gold layers, which was constructed with Visual Molecular Dynamics (VMD).^[^ ^22^ ^]^ The BB‐peptides were grafted at X‐Y plane to ensure a density of 6.5%. Polymers and peptides were connected through the sulfur (S) atoms of cysteine side chains, which replaced the bromine (Br) atoms at the terminal positions of the polymers. A second gold‐polymer‐peptide structure was duplicated, inverted about the origin, and positioned such that the distance between the gold surface was 9 nm along the Z‐axis, with peptides orientation inward. Grafting density was controlled by adjusting the spacing between side chains (with and without peptides) on the X‐Y plane.

### Molecular Dynamics and Quantum Mechanics Simulations—Molecular Dynamics (MD) Simulations and Umbrella Sampling

The pulling MD simulations were performed by the Gromacs software package^[^ ^23^ ^]^ with the Amber ff14SB force field.^[^ ^24^ ^]^ Topology parameters for gold atoms were derived from a reference study,^[^ ^25^ ^]^ while polymer unit topologies were generated using the Sobtop 1.0(dev5) program.^[^ ^26^ ^]^ Atomic restricted electrostatic potential (RESP) charges^[^ ^27^ ^]^ were calculated with Gaussian^[^ ^28^ ^]^ at the B3LYP/def2‐SVP level^[^ ^29^ ^]^ and fitted using Multiwfn.^[^ ^30^ ^]^ The Y^3+^ ion parameter was obtained from the UFF force field.^[^ ^31^ ^]^

Based on each constructed BB‐Pep system, we first extended each system by 20 nm along the Z‐axis and added inside K^+^ or Y^3+^ ions (equimolar to peptides) and Cl^−^ counterions for neutralization; Then, each system was energy‐minimized for 2000 steps via the steepest descent algorithm, and pushed together in Z‐directions at 0.001 nm ps^−1^ for 100 000 steps, to ensure peptides fully contacted with ions; Then the inter‐surface gap was solvated with TIP3P water^[^ ^32^ ^]^ and equilibrated for 2000 ps at 298.15 K under an NVT ensemble using the velocity‐rescale thermostat.^[^ ^33^ ^]^ Finally, pulling dynamics were conducted in an NVE ensemble by displacing the gold layers in opposite Z‐directions at 0.001 nm ps^−1^. Forces and displacements were averaged over 1000 steps and recorded.

Frames from the pulling trajectory were extracted at 0.05 nm intervals along the Z‐direction, defining overlapping umbrella sampling windows. Each window underwent a 100 ps NPT equilibration (298.15 K, 1 bar), followed by a 10 ns production phase simulation. The weighted histogram analysis method (WHAM)^[^ ^34^ ^]^ was used to compute the PMF profile via the *gmx wham* tool^44^.

### Molecular Dynamics and Quantum Mechanics Simulations—MD Trajectory Analyzation

Data analyzation was performed on the pulling MD trajectory, using custom Python scripts based on the MDAnalysis package.^[^ ^35^ ^]^ The π–π and cation‐π interactions in each system were determined using MDAnalysis by detecting π–π stacking between phenyl rings in the two BB‐Pep layers, and cation‐π stacking between phenyl rings and ions (including NH_3_^+^, K^+^, and Y^3+^).

The phenyl ring contacts were considered existence if the distance between any atom pair was less than 4.5 Å. The phenyl ring‐cation contacts were considered existence if the distance between the cation and the center of the phenyl ring was less than 10 Å. The 2D distribution of π–π stacking was calculated based on the centroid distance between two aromatic rings and the angle between their aromatic normal vectors (using the supplementary angle if the angle exceeded 90°). The 2D distribution was determined by kernel density estimation. The structure and trajectory were visualized in VMD.^[^ ^22^ ^]^

### Molecular Dynamics and Quantum Mechanics Simulations—Quantum Mechanical Analysis

Quantum Mechanical calculations were performed with the Gaussian 16 software package.^[^ ^36^ ^]^ The side chains of particular phenyl‐cation‐phenyl configurations from MD conformations were extracted and capped with H atoms, with Cβ atoms fixed during geometric optimization at the B3LYP/def2‐SV(P) level^[^ ^29^ ^]^ with GD3 empirical dispersion^[^ ^37^ ^]^ and SMD implicit solvation model. Subsequent single‐point energy calculations employed the def2‐SVP basis set.^[^ ^29b^ ^]^ Weak interactions were evaluated via the Integrated Gradient Model based on Hirshfeld partitioning (IGMH)^[^ ^38^ ^]^ using Multiwfn^[^ ^30^ ^]^ and visualized with VMD.^[^ ^22^ ^]^

## Conflict of Interest

The authors declare no conflict of interest.

## Supporting information



Supporting Information

## Data Availability

The data that support the findings of this study are available from the corresponding author upon reasonable request.
