# Supplementary material for: Ion Valency as a Molecular Switch for Salt‐Resistant Underwater Adhesion
Source: Adv Mater. 2025 Aug 5;37(42):e08666. doi: 10.1002/adma.202508666 (PMC12548505; doi:10.1002/adma.202508666)
Supplement: Supplementary file 1 — Supporting Information [file ADMA-37-e08666-s001.docx]

Supporting Information

Ion Valency as a Molecular Switch for Salt-Resistant Underwater Adhesion

*Chang-Sheng Wang,^a†^ Jiaxing Zhang,^bc†^ Hu Zhang,^a^ Wojciech Raj,^a^ Nahid Hassanpour,^a^ Duy Anh Pham,^d^ Hui Guo, ^a^ Xingxun Liu,^e^ Heng Chang,^f^ Alexandre A. Arnold,^g^ Isabelle Marcotte,^g^ Rongxin Su,^bcf^ Wei Qi,^bc^ Xavier Banquy ^adh*^*

1. **Materials and Methods**

1.1 Materials

Triethylamine (TEA) was dried with calcium hydride and freshly distilled every time before use. 2-(Trimethylsilyloxy)ethyl methacrylate (HEMA-TMS), 2-(dimethylamino)ethyl methacrylate (DMAEMA), methyl methacrylate (MMA), poly(ethylene glycol) methyl ether methacrylate (PEGMA, average *M*_n_ 300) were passed through a basic Al_2_O_3_ column to remove inhibitor. Potassium fluoride, 2,6-di-tertbutylphenol, 2-bromoisobutyryl bromide (BIBB), 4,4′-dinonyl-2,2′-dipyridyl (dNbpy), copper (II) bromide (CuBr_2_), copper(I) bromide (CuBr), tributyltin hydride, tetra-n-butylammonium fluoride (TBAF), potassium carbonate (K2CO3), bromoethane, cysteamine hydrochloride, N-hydroxysuccinimide (NHS) and sodium bicarbonate were used without further purification. Ethylene bis(2-bromoisobutyrate) (2f-BIB) were synthesized according to the following reference.^[1]^ All chemicals were purchased from Sigma-Aldrich unless otherwise stated.

**2. Bottlebrush polymers synthesis strategy**

2.1 Synthesis of bottlebrush polymers

The macro-initiators for synthesizing BB-PEGMA were prepared according to our previous work.^[39]^

PEGMA polymerization for BB-PEGMA was carried out using the ATRP method. A dry 15 mL round-bottom flask was charged with CuBr (4.9 mg, 0.0347 mmol), CuBr₂ (0.86 mg, 0.0038 mmol), and dNbpy (31.5 mg, 0.0772 mmol). The flask was sealed with a stopper and covered with black tape. Separately, BB-Br macroinitiator (15 mg, 90 nmol) and PEGMA (3.47 g, 11.58 mmol) were dissolved in anisole (5.0 mL) under stirring for several minutes. A stainless-steel cannula connected the flask and vial, and the solution was degassed using four freeze-pump-thaw cycles. On the final cycle, the flask was warmed to room temperature and filled with argon. The system was briefly placed under vacuum, then switched to argon to transfer the solution from the vial to the flask. Once transferred, the cannula was removed, and the flask was sealed tightly. The mixture was heated in an oil bath at 50 °C for 3 hours, then quenched by exposure to air. The resulting polymer was purified by dialysis in methanol and stored in methanol at 4 °C.


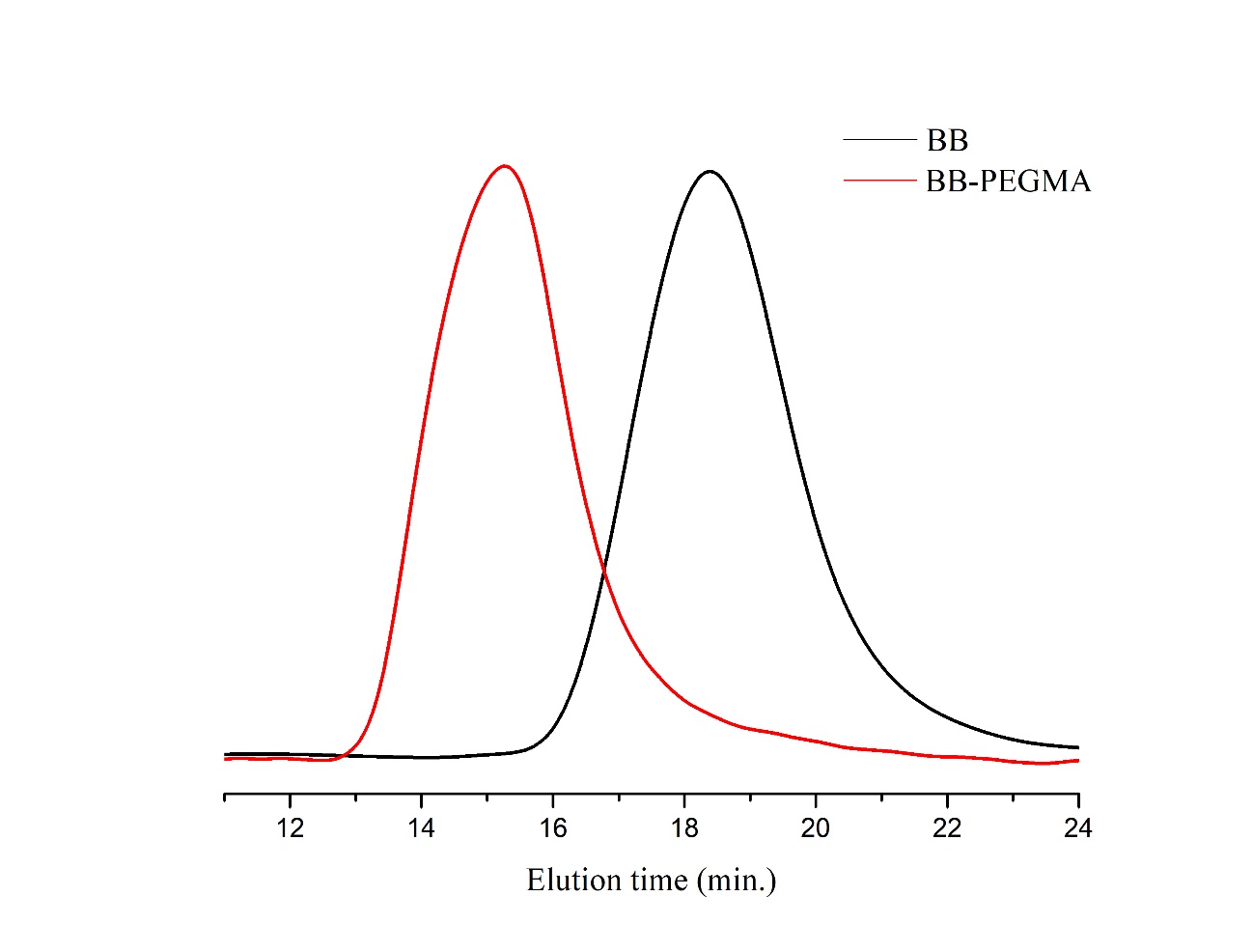


Figure S1: GPC curves of synthesized BB macroiniator and BB-PEGMA bottlebrush polymer

2.2 Synthesis of BB-PEGMA-Pep and BB-PEGMA-Control peptide

BB polymer (123.20 mg in methanol) was dialyzed against DMF and transferred to a 10 mL flask. K₂CO₃ (0.30 mg) was added to the polymer solution, followed by the addition of certain amount of adhesive peptide or control peptide with stirring (Table S1). The reaction was allowed to proceed overnight at room temperature. The final product was purified by dialysis, first against DMF and then against water, and stored in water.

Table S1 Feeding ratio and actual ratio determined by NMR

|  | Peptide added (mg) | In feed ratio (%) | Ratio in composition calculated by NMR (%) |
| --- | --- | --- | --- |
| BB-Pep6.5 | 0.31 | 6.5 | 3.6 |
| BB-Pep13 | 0.62 | 13.0 | 9.1 |
| BB-Pep26 | 1.24 | 26 | 24.1 |

**3. Calculation of peptide in composition of BB by NMR spectrum**


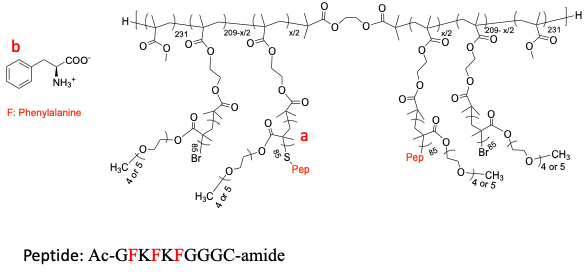


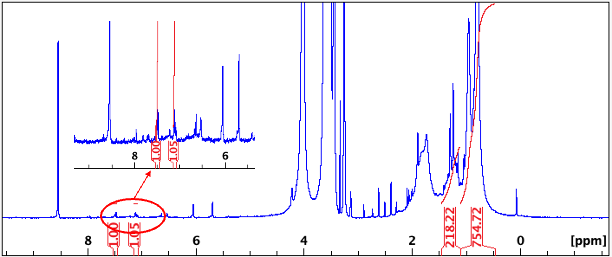


Figure S2: ^1^H solution NMR spectrum of BB-Pep6.5 recorded in CDCl_3_.

The peptide ratio in the composition was set to X%. Based on the integral of the NMR spectrum, the following equation was derived:

$$\frac{a}{b}= \frac{209\times2\times85\times3}{209\times2\times x\%\times3\times5}=\frac{(218.2+754.7)}{(1.00+1.05)}$$

Similarly, *x* % values for BB-Pep13 and BB-Pep26 were determined using the same method (Table S1), based on the integral of the peaks shown in Figures S3 and S4.


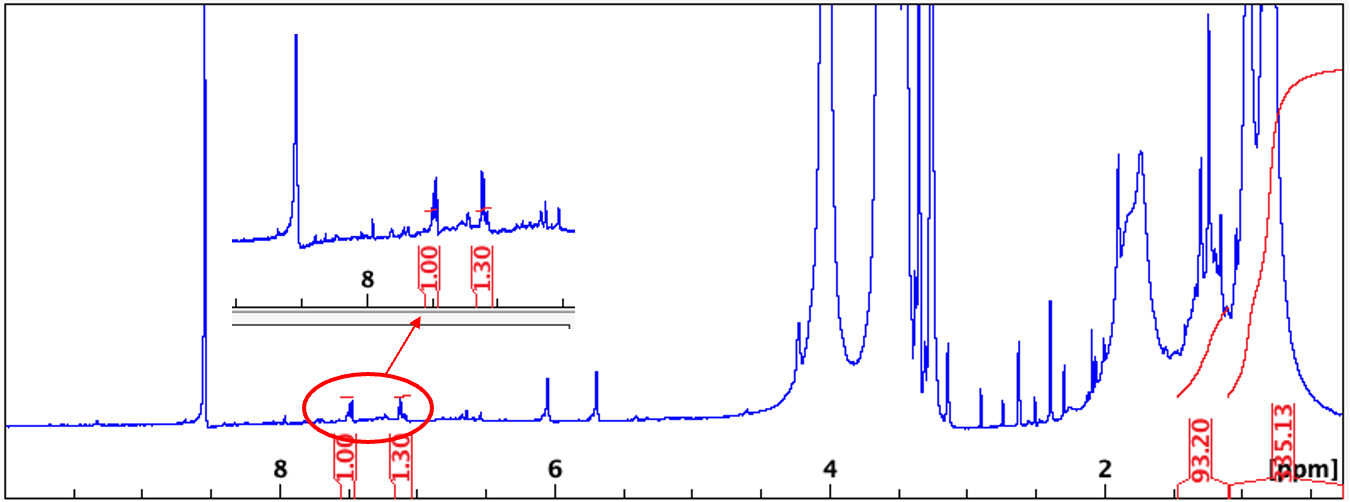


Figure S3: ^1^H solution NMR spectrum of BB-Pep13 recorded in CDCl_3_.


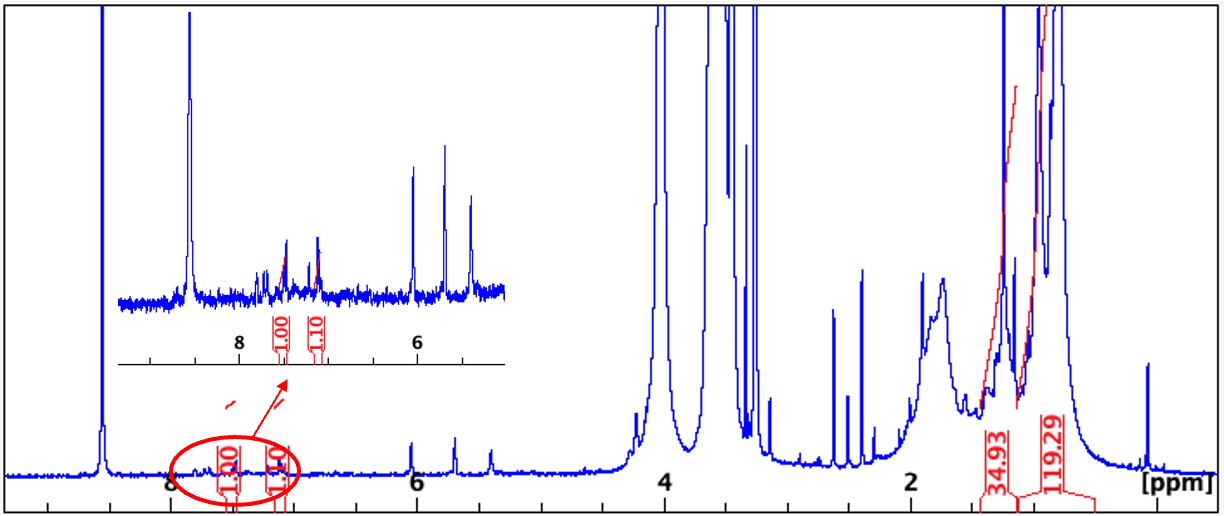


Figure S4: ^1^H solution NMR spectrum of BB-Pep26 recorded in CDCl_3_.

Figure S5 Cohesion comparison between BB-control (Ac-GLKLKLGGGC-amide) and BB-Pep (Ac-GFKFKFGGGC-amide) films at 13% grafting density, prepared at 50 μg/mL.

**4. Dynamic light scattering (DLS)**

Hydrodynamic diameters and polydispersity indices (PDI) of the samples were determined using a Malvern Zetasizer Nano ZS (Malvern Panalytical, UK) equipped with a 173° backscatter detector. Measurements were conducted at 25°C following a 2-minute equilibration period. Samples were analyzed in triplicate (three consecutive measurements per run) to ensure reproducibility. Data was processed using Zetasizer Software v7.13, with intensity-weighted distributions reported.


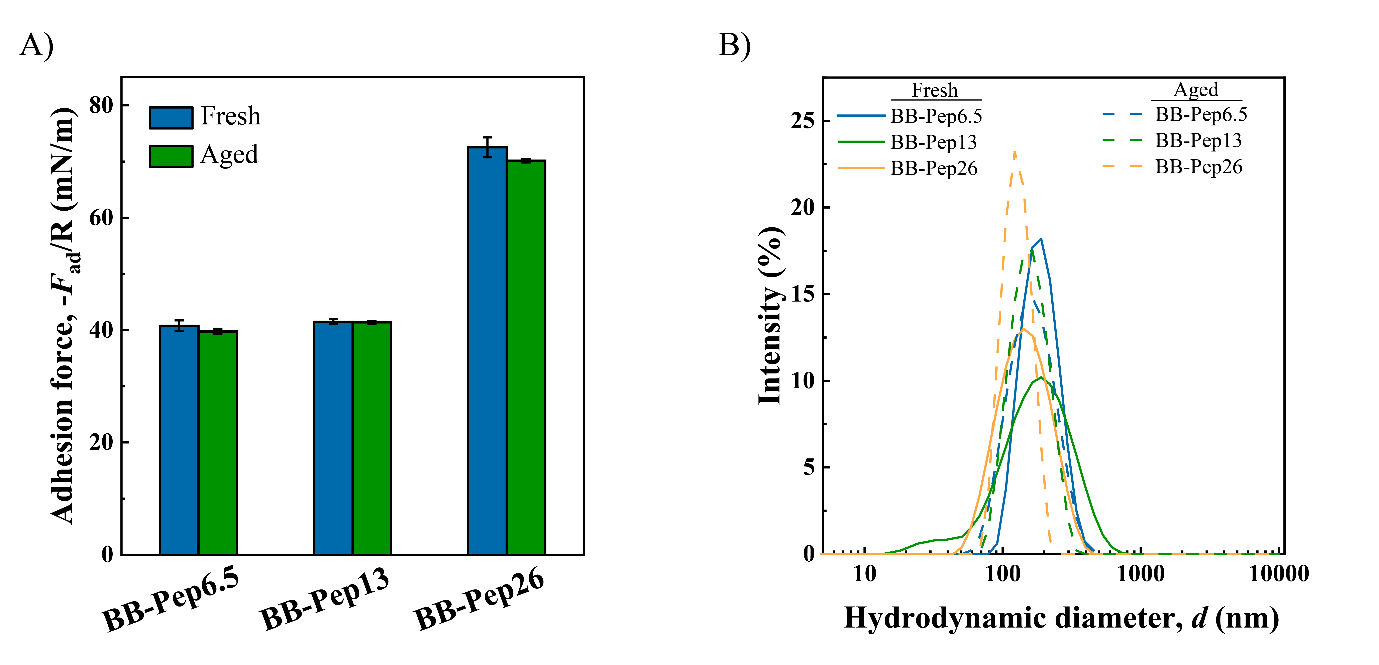


Figure S6 A) Comparison of adhesion energy between fresh samples and samples aged for 9 months at 4°C. B) Size distribution (e.g., hydrodynamic diameter) of fresh samples versus those stored at room temperature (RT) for 14 days, assessed via DLS.

We also evaluated the stability of BB-Pep samples under different storage conditions. Adhesion measurements revealed no significant difference between freshly prepared samples and those stored for nine months at 4°C, regardless of grafting density (Figure S5A), demonstrating excellent storage stability. Additionally, DLS analysis showed that the polymer size remained constant after 14 days of storage at room temperature (Figure S5B). This indicates that intermolecular interactions in solution are too weak to drive polymer aggregation.

**5. NMR spectrum**


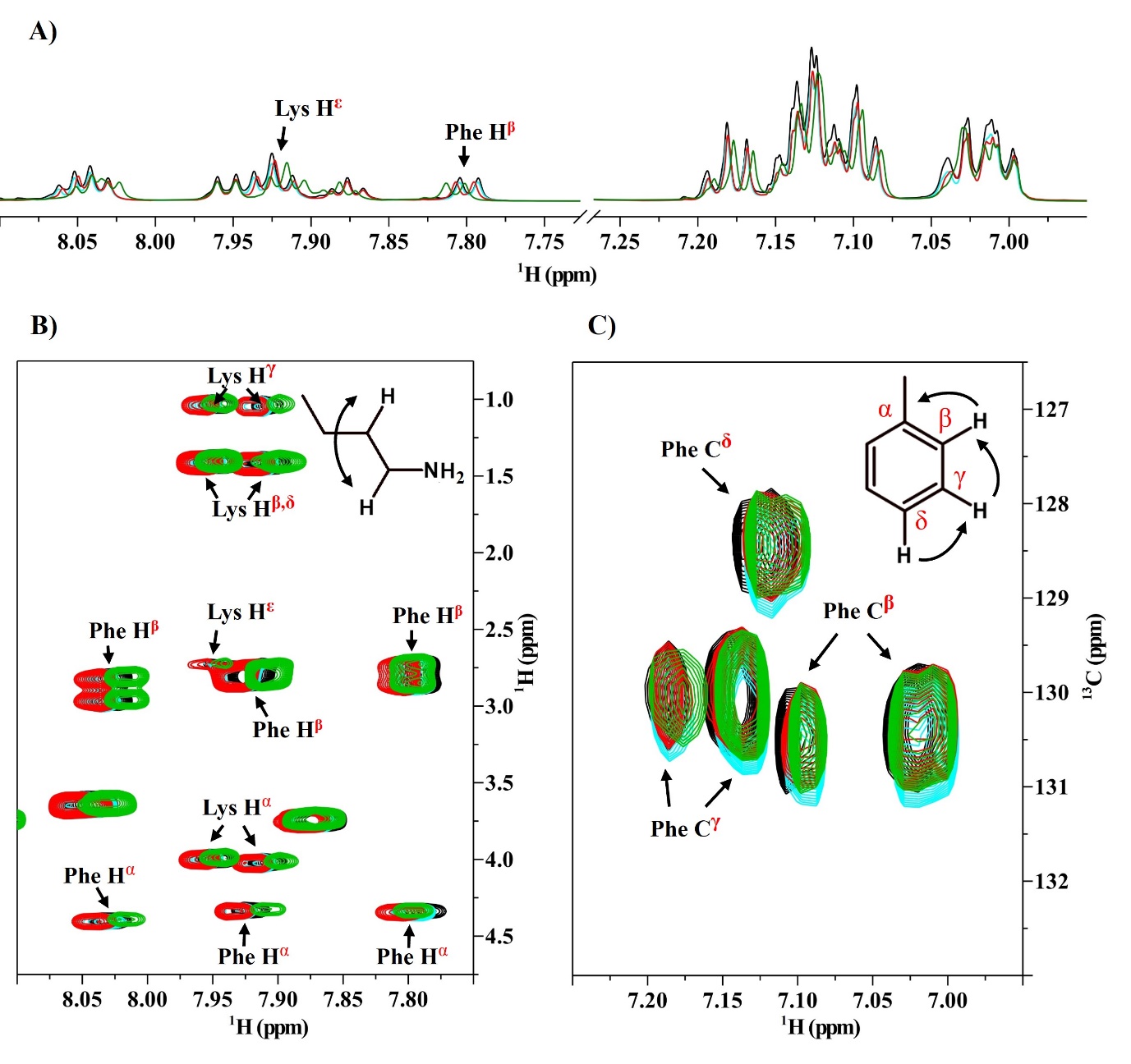


Figure S7. Solution NMR determines the molecular mechanism of π-interaction forming and disruption in phenylalanine and lysine upon exposure for KCl. **A)** The one-dimensional ^1^H NMR spectrum of the Ac-GFKFKFGGGC-amide peptide (5 mmol, black) in the presence of increasing concentrations of monovalent salt KCl (1mmol – cyan, 10 mmol – red, and 100 mmol – green) in D_2_O:DMSO-*d*_6_ (9:1 v/v; 300K). **B)** Expanded region of the 2D ^1^H-TOCSY spectrum showing the correlations between lysine H^α^–H^ε^ protons and phenylalanine H^α^–H^β^ protons. **C)** Aromatic regions of the 2D ^1^H-^13^C-HSQC spectrum displaying proton-carbon correlations. Arrows indicate chemical shift differences at the aromatic ring.


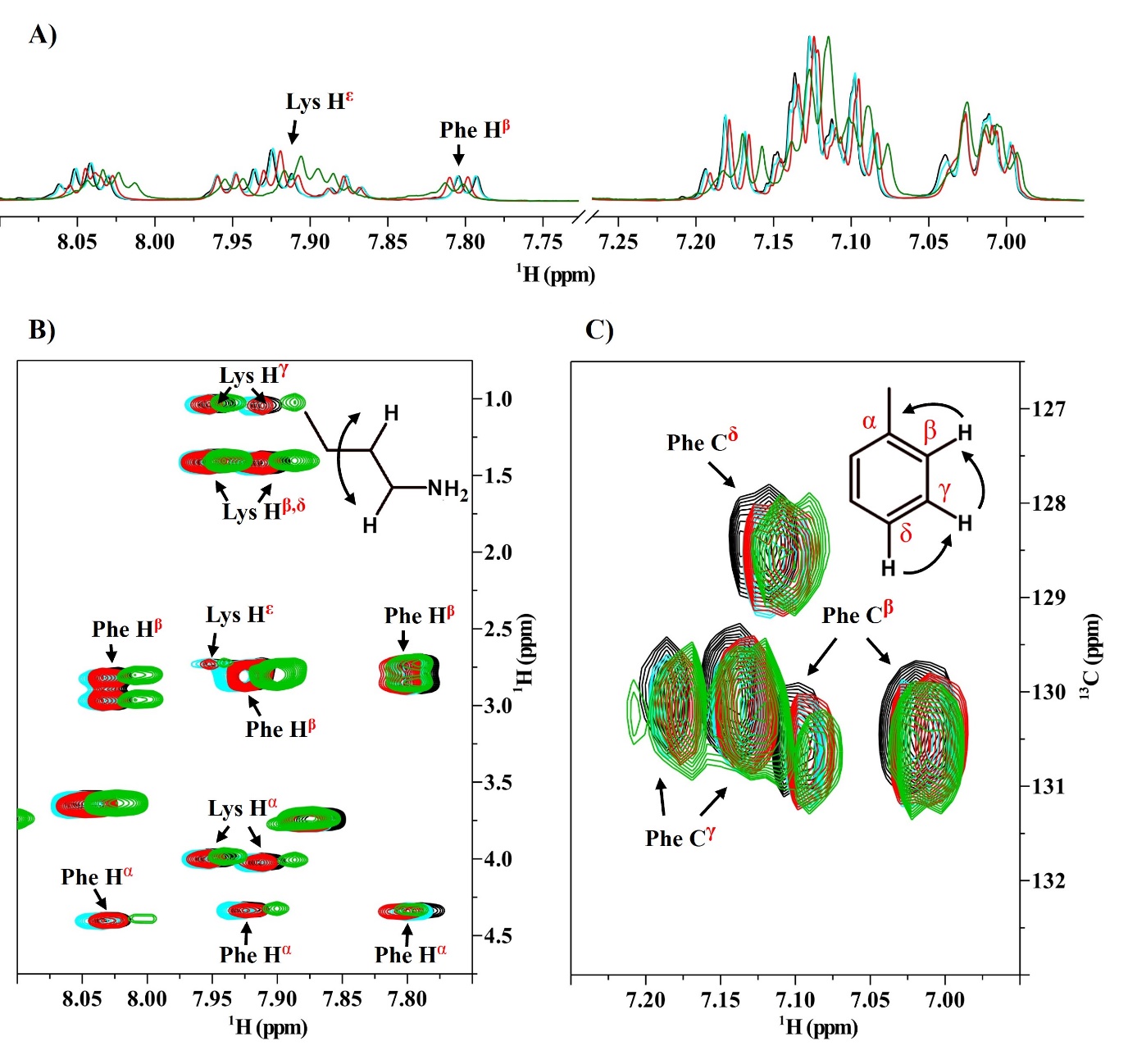


Figure S8. Solution NMR determines the molecular mechanism of π-interaction forming and disruption in phenylalanine and lysine upon exposure for YCl_3_. **A)** The one-dimensional ^1^H NMR spectrum for the Ac-GFKFKFGGGC-amide peptide (5 mmol, black) in the presence of increasing concentrations of trivalent salt YCl_3_ (1mmol – cyan, 10 mmol – red, and 100 mmol – green) in D_2_O:DMSO-*d*_6_ (9:1 v/v; 300K). **B)** Expanded region of the 2D ^1^H-TOCSY spectrum showing the correlations between lysine H^α^–H^ε^ protons and phenylalanine H^α^–H^β^ protons. **C)** Aromatic regions of the 2D ^1^H-^13^C-HSQC spectrum displaying proton-carbon correlations. Arrows indicate chemical shift differences at the aromatic ring.

Table S2. Characteristic ^1^H chemical shift variations of Phenylalanine (Phe) and Lysine (Lys) residues of the adhesive peptide upon addition of cationic salts KCl and YCl_3_. The H^α^ ^1^H chemical shift variation of glycine is shown for comparison.

| Assignement | KCl | | | YCl_3_ | | |
| --- | --- | --- | --- | --- | --- | --- |
|  | 1 mM | 10 mM | 100 mM | 1 mM | 10 mM | 100 mM |
| Phe NH | -0.0003 | -0.0027 | -0.0087 | -0.0008 | -0.0059 | -0.0091 |
| Phe NH | 0.0000 | 0.0019 | 0.0096 | 0.001 | 0.0060 | 0.0194 |
| Phe NH | 0.0000 | 0.0005 | 0.0073 | 0.0006 | 0.0031 | 0.0172 |
| Phe γ | 0.0000 | 0.0005 | 0.0042 | 0.0003 | 0.0028 | 0.0113 |
| Phe β | 0.0000 | 0.0008 | 0.0012 | 0.0002 | 0.0050 | 0.0075 |
| Phe δ | 0.0000 | 0.0006 | 0.0029 | 0.0003 | 0.0022 | 0.0092 |
| Lys NH | 0.0001 | 0.0012 | 0.0076 | 0.0007 | 0.0041 | 0.0171 |
| Lys NH | 0.0000 | 0.0000 | 0.0000 | 0.0000 | 0.0005 | 0.0166 |

Figure S9. Characteristic ^1^H chemical shift variations of Phenylalanine (Phe) and Lysine (Lys) residues of the adhesive peptide upon addition of cationic salts KCl and YCl_3_. Note that the changes in chemical shift increase with salt concentration for both K^+^ and Y^3+^ and that the changes induced by Y^3+^ are systematically larger than those induced by K^+^.

**6. Derivation of the** **thermodynamic mass-balance model**

The model considers that monovalent and multivalent ions influence the specific pairing process between peptides through the following equilibria:

$$\begin{aligned} P+M\leftrightarrow PM\#\left( a \right) \end{aligned}$$

$$\begin{aligned} P+T\leftrightarrow PT\#\left( b \right) \end{aligned}$$

$$\begin{aligned} P+PT\leftrightarrow P_{2}T\#\left( c \right) \end{aligned}$$

$$\begin{aligned} P+P\leftrightarrow P_{2}\#\left( d \right) \end{aligned}$$

where *P* represents the peptide, *M* the monovalent ion K^+^, and *T* the multivalent ion, specifically trivalent ion Y^3+^. The model rests on two critical assumptions: (1) all surface-bound peptides are exclusively available for inter-plane pairing, neglecting competitive intra-plane interactions, this point will be discussed later in our analysis of the limitations of the model, and (2) adhesion arises solely from π-π, cation-π and π-cation-π interactions, omitting contributions from secondary forces such as hydrogen bonding and van der Waals interactions. In the present model, adhesion is governed by the formation of peptide-peptide pairing between the surfaces, and therefore by the concentrations of $P_{2}$ and π-cation-π complexes $P_{2}T$.

To capture the interplay between ion valency and ionic strength, different apparent binding constants for each equilibrium reaction were defined as:

$$\begin{aligned} K_{1}= \frac{[PM]}{\left[ P \right][M]}\#\left( 1 \right) \end{aligned}$$

$$\begin{aligned} K_{2}= \frac{[PT]}{\left[ P \right][T]}\#\left( 2 \right) \end{aligned}$$

$$\begin{aligned} K_{3}= \frac{[P_{2}T]}{\left[ P \right][PT]}\#\left( 3 \right) \end{aligned}$$

$$\begin{aligned} K_{4}= K_{2}K_{3}=\frac{\left[ P_{2}T \right]}{\left[ P \right]^{2}\left[ T \right]}\#\left( 4 \right) \end{aligned}$$

$$\begin{aligned} K_{5}= \frac{\left[ P_{2} \right]}{\left[ P \right]^{2}}\#\left( 5 \right) \end{aligned}$$

Substituting Eq. (1)-(5) into the conservation equation:

$$\begin{aligned} \left[ P \right]_{T}=[P]+\left[ PM \right]+\left[ PT \right]+2\left[ P_{2}T \right]+2[P_{2}]\#\left( 6 \right) \end{aligned}$$

where [*P*]_T_ is the total concentration of peptide adsorbed on the surfaces.

$$\begin{aligned} \left[ P \right]_{T}-2\left( \left[ P_{2}T \right]+[P_{2}] \right)=K_{1}\left[ P \right]\left[ M \right]+K_{2}\left[ P \right]\left[ T \right]+\left[ P \right]\#\left( 7 \right) \end{aligned}$$

$$\begin{aligned} \left[ P \right]=\frac{\left[ P \right]_{T}-2\left( \left[ P_{2}T \right]+[P_{2}] \right)}{K_{1}\left[ M \right]+K_{2}\left[ T \right]+1}\#\left( 8 \right) \end{aligned}$$

From eq 4 and eq 5, we have

$$\begin{aligned} \left[ P_{2}T \right]+\left[ P_{2} \right]=K_{4}\left[ T \right]\left[ P \right]^{2}+K_{5}[P]^{2}=\frac{K_{4}\left[ T \right]+K_{5}}{\left( K_{1}[M]+K_{2}\left[ T \right]+1 \right)^{2}}\left[ \left[ P \right]_{T}-2\left( \left[ P_{2}T \right]+{[P}_{2}] \right) \right]^{2}\#\left( 9 \right) \end{aligned}$$

$$\begin{aligned} \frac{\left[ P_{2}T \right]}{\left[ P \right]_{T}}+\frac{\left[ P_{2} \right]}{\left[ P \right]_{T}}=\frac{(K_{4}\left[ T \right]+K_{5})\left[ P \right]_{T}}{\left( K_{1}[M]+K_{2}\left[ T \right]+1 \right)^{2}}\left[ 1-2(\frac{\left[ P_{2}T \right]}{\left[ P \right]_{T}}+\frac{\left[ P_{2} \right]}{\left[ P \right]_{T}}) \right]^{2}\#\left( 10 \right) \end{aligned}$$

$$\begin{aligned} \left[ P_{2}T \right]-\left[ P_{2} \right]=K_{4}\left[ T \right]\left[ P \right]^{2}-K_{5}[P]^{2}=\frac{K_{4}\left[ T \right]-K_{5}}{\left( K_{1}[M]+K_{2}\left[ T \right]+1 \right)^{2}}\left[ \left[ P \right]_{T}-2\left( \left[ P_{2}T \right]+{[P}_{2}] \right) \right]^{2}\#\left( 11 \right) \end{aligned}$$

$$\begin{aligned} \frac{\left[ P_{2}T \right]}{\left[ P \right]_{T}}-\frac{\left[ P_{2} \right]}{\left[ P \right]_{T}}=\frac{\left( K_{4}\left[ T \right]-K_{5} \right)\left[ P \right]_{T}}{\left( K_{1}[M]+K_{2}\left[ T \right]+1 \right)^{2}}\left[ 1-2\left( \frac{\left[ P_{2}T \right]}{\left[ P \right]_{T}}+\frac{\left[ P_{2} \right]}{\left[ P \right]_{T}} \right) \right]^{2}\#\left( 12 \right) \end{aligned}$$

Let

$$\begin{aligned} \frac{\left[ P_{2}T \right]}{\left[ P \right]_{T}}=x and \frac{\left[ P_{2} \right]}{\left[ P \right]_{T}}=y\#\left( 13 \right) \end{aligned}$$

And

$$\begin{aligned} B= \frac{\left( K_{4}\left[ T \right]+K_{5} \right)\left[ P \right]_{T}}{\left( K_{1}\left[ M \right]+K_{2}\left[ T \right]+1 \right)^{2}}\#\left( 14 \right) \end{aligned}$$

$$\begin{aligned} C= \frac{\left( K_{4}\left[ T \right]-K_{5} \right)\left[ P \right]_{T}}{\left( K_{1}\left[ M \right]+K_{2}\left[ T \right]+1 \right)^{2}}\#\left( 15 \right) \end{aligned}$$

Then eq 10 and eq 12 becomes

$$\begin{aligned} x+y=B\left( 1-2\left( x+y \right) \right)^{2}=B\left( 1+4x^{2}+4y^{2}-4\left( x+y \right)+8xy \right)\#\left( 16 \right) \end{aligned}$$

$$\begin{aligned} x-y=C\left( 1-2\left( x+y \right) \right)^{2}=C\left( 1+4x^{2}+4y^{2}-4\left( x+y \right)+8xy \right)\#\left( 17 \right) \end{aligned}$$

$x\ll1 and y\ll1,$ ${so x}^{2}\approx0$, $y^{2}\approx0, and xy\approx0$ eq 16 is simplified as

$$\begin{aligned} x+y=\frac{B}{1+4B}\#\left( 18 \right) \end{aligned}$$

When $B\gg1, x+y=\frac{1}{4}$, this contradicts with $x+y\ll1$.

When $B\ll1, x+y=B$, in consistent with $x+y\ll1$

That is to say, eq 18 becomes

$$\begin{aligned} x+y=B\#\left( 19 \right) \end{aligned}$$

Substitute eq 19 to eq 17, we get

$$\begin{aligned} x-y=C\left( 1-4B \right)\#\left( 20 \right) \end{aligned}$$

Using eq 19 and eq 20, we can get

$$\begin{aligned} x=\frac{B+C}{2}-2BC=\frac{K_{4}\left[ T \right]\left[ P \right]_{T}}{\left( K_{1}\left[ M \right]+K_{2}\left[ T \right]+1 \right)^{2}}-\frac{2(\left( K_{4}\left[ T \right]+K_{5} \right)\left( K_{4}\left[ T \right]-K_{5} \right)\left[ P \right]_{T}^{2}}{\left( K_{1}\left[ M \right]+K_{2}\left[ T \right]+1 \right)^{4}}\#\left( 21 \right) \end{aligned}$$

$$\begin{aligned} y=\frac{B-C}{2}+2BC=\frac{K_{5}\left[ P \right]_{T}}{\left( K_{1}\left[ M \right]+K_{2}\left[ T \right]+1 \right)^{2}}+\frac{2(\left( K_{4}\left[ T \right]+K_{5} \right)\left( K_{4}\left[ T \right]-K_{5} \right)\left[ P \right]_{T}^{2}}{\left( K_{1}\left[ M \right]+K_{2}\left[ T \right]+1 \right)^{4}}\#\left( 22 \right) \end{aligned}$$

Or

$$\begin{aligned} \left[ P_{2}T \right]=\frac{K_{4}\left[ T \right]\left[ P \right]_{T}^{2}}{\left( K_{1}\left[ M \right]+K_{2}\left[ T \right]+1 \right)^{2}}-\frac{2(\left( K_{4}\left[ T \right]+K_{5} \right)\left( K_{4}\left[ T \right]-K_{5} \right)\left[ P \right]_{T}^{3}}{\left( K_{1}\left[ M \right]+K_{2}\left[ T \right]+1 \right)^{4}}\#\left( 23 \right) \end{aligned}$$

$$\begin{aligned} \left[ A_{2} \right]=\frac{K_{5}\left[ P \right]_{T}^{2}}{\left( K_{1}\left[ M \right]+K_{2}\left[ T \right]+1 \right)^{2}}+\frac{2(\left( K_{4}\left[ T \right]+K_{5} \right)\left( K_{4}\left[ T \right]-K_{5} \right)\left[ P \right]_{T}^{3}}{\left( K_{1}\left[ M \right]+K_{2}\left[ T \right]+1 \right)^{4}}\#\left( 24 \right) \end{aligned}$$

$$\begin{aligned} F_{ad}\sim\left( \left[ P_{2}T \right]+\left[ P_{2} \right] \right)\delta=\frac{\left( K_{4}\left[ T \right]+K_{5} \right){[P]}_{T}^{2}\delta}{\left( K_{1}\left[ M \right]+K_{2}\left[ T \right]+1 \right)^{2}}\#\left( 25 \right) \end{aligned}$$

In the case of no ions ($\left[ P_{2}T \right]=0, \left[ M \right]=0$ and [*T*] = 0), the adhesion is predicted by the following equation:

$$\begin{aligned} F_{ad}\sim\delta_{y}\left[ P_{2} \right]={\delta_{y}K}_{5}\left[ P \right]_{T}^{2}\#\left( 26 \right) \end{aligned}$$

**Extended thermodynamic mass-balance model**

If taking the intra-plane into consideration, we have to introduce an intra-plane peptide pairing reaction within a single plane:

$$\begin{aligned} P+P\leftrightarrow P_{2, intra}\#\left( e \right) \end{aligned}$$

The corresponding equilibrium constant can be defined as:

$$\begin{aligned} K_{6}= \frac{\left[ P_{2, intra} \right]}{\left[ P \right]^{2}}\#\left( 27 \right) \end{aligned}$$

The conservation equation becomes:

$$\begin{aligned} \left[ P \right]_{T}=\left[ P \right]+\left[ PM \right]+\left[ PT \right]+2\left[ P_{2}T \right]+2\left[ P_{2} \right]+2[P_{2,intra}]\#\left( 28 \right) \end{aligned}$$

Under the conditions $\frac{{[P}_{2}T]}{[{P]}_{T}}\ll1$ and $\frac{{[P}_{2}]}{[{P]}_{T}}\ll1$

The conservation equation can be simplified as:

$$\begin{aligned} \left[ P \right]_{T}=\left[ P \right]+\left[ PM \right]+\left[ PT \right]+2[P_{2,intra}]\#\left( 29 \right) \end{aligned}$$

Substitute the expressions for each species using the binding constants:

$$\begin{aligned} \left[ P \right]_{T}={\left[ P \right]+K}_{1}\left[ P \right]\left[ M \right]+K_{2}\left[ P \right]\left[ T \right]+2K_{6}[P]^{2}\#\left( 30 \right) \end{aligned}$$

$$\begin{aligned} \left[ P \right]_{T}={\left[ P \right](1+K}_{1}\left[ M \right]+K_{2}\left[ T \right])+2K_{6}\left[ P \right]^{2}\#\left( 31 \right) \end{aligned}$$

Define:

$$\begin{aligned} a= {1+K}_{1}\left[ M \right]+K_{2}\left[ T \right]\#\left( 32 \right) \end{aligned}$$

The conservation equation is now:

$$\begin{aligned} \left[ P \right]_{T}=a[P]+{2K_{6}\left[ P \right]}^{2}\#\left( 33 \right) \end{aligned}$$

Then we have

$$\begin{aligned} \left[ P \right]=\frac{[{P]}_{T}}{a+2K_{6}\left[ P \right]}\#\left( 34 \right) \end{aligned}$$

For small $2K_{6}[P]$, we have

$$\begin{aligned} \left[ P \right]=\frac{[P]_{T}}{a}\cdot\frac{1}{\left( 1+\frac{2K_{6}\left[ P \right]_{T}}{a^{2}} \right)}\#\left( 35 \right) \end{aligned}$$

Then

$$\begin{aligned} \left[ P_{2}T \right]+\left[ P_{2} \right]=\frac{{(K}_{4}\left[ T \right]+K_{5})P_{T}^{2}}{a^{2}}\cdot\frac{1}{\left( 1+\frac{2K_{6}\left[ P \right]_{T}}{a^{2}} \right)^{2}}\#\left( 36 \right) \end{aligned}$$

or

$$\begin{aligned} \left[ P_{2}T \right]+\left[ P_{2} \right]=\frac{{(K}_{4}\left[ T \right]+K_{5})[{P]}_{T}^{2}}{{({1+K}_{1}\left[ M \right]+K_{2}\left[ T \right])}^{2}}\cdot\frac{1}{\left( 1+\frac{2K_{6}\left[ P \right]_{T}}{{({1+K}_{1}\left[ M \right]+K_{2}\left[ T \right])}^{2}} \right)^{2}}\#\left( 37 \right) \end{aligned}$$

In the case of no ions ($\left[ P_{2}T \right]=0, \left[ M \right]=0$ and [*T*] = 0), the adhesion is predicted by the following equation:

$$\begin{aligned} F_{ad}\sim\delta_{y}\left[ P_{2} \right]=\frac{{\delta_{y}K}_{5}\left[ P \right]_{T}^{2}}{(1+2K_{6}{[P]}_{T})^{2}}\#\left( 38 \right) \end{aligned}$$

**7. Surface coverage determination**

Surface coverage was measured by analyzing AFM images using ImageJ. The images were first converted to 8-bit format, followed by adjustments to brightness and contrast. They were then inverted and thresholded, resulting in BB-Pep chains appearing as black features. To better isolate the chains, median and minimum filters were applied. The number of polymer chains was determined using the particle analysis function. Small particles not corresponding to BB-Pep chains were excluded by setting an appropriate size threshold. The surface coverage is determined using the equation:

$$\sigma=\frac{Number of peptides}{area} =\frac{number of polymer chains \times number of repeating unit \times actual grafting density}{area}$$

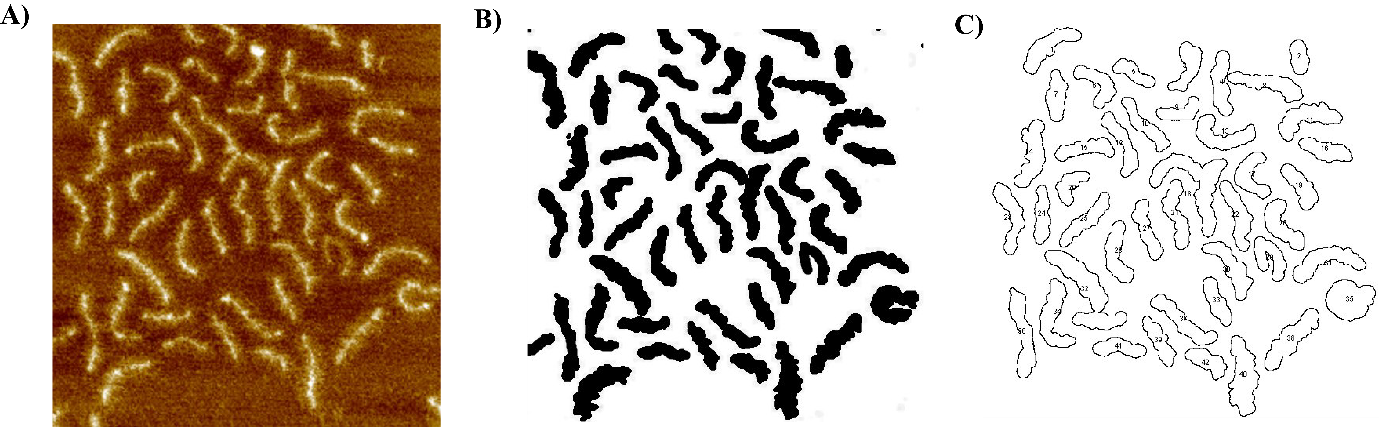


Figure S10 Surface coverage determination. **A)** AFM images; **B)** the images after treatment; **C)** Counting of BB-Pep chains

**8. Molecular dynamics**


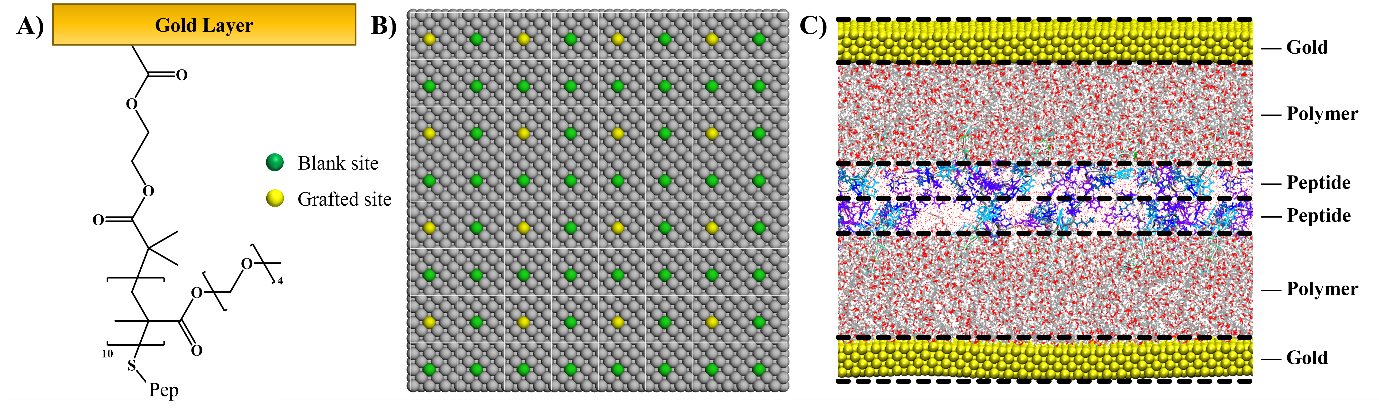


Figure S11 **A)** Model system construction of BB-Pep system. The main chain of polymer was simplified as a gold layer, with the side chain with peptide grafted. **B)** The top view scheme of the gold layer, with possible graft site colored green/yellow. Green: not grafted sites (Br); Yellow: Grafted sites (S); White: Background (Au). **C)** The side view of gold-polymer-peptide initial structure.


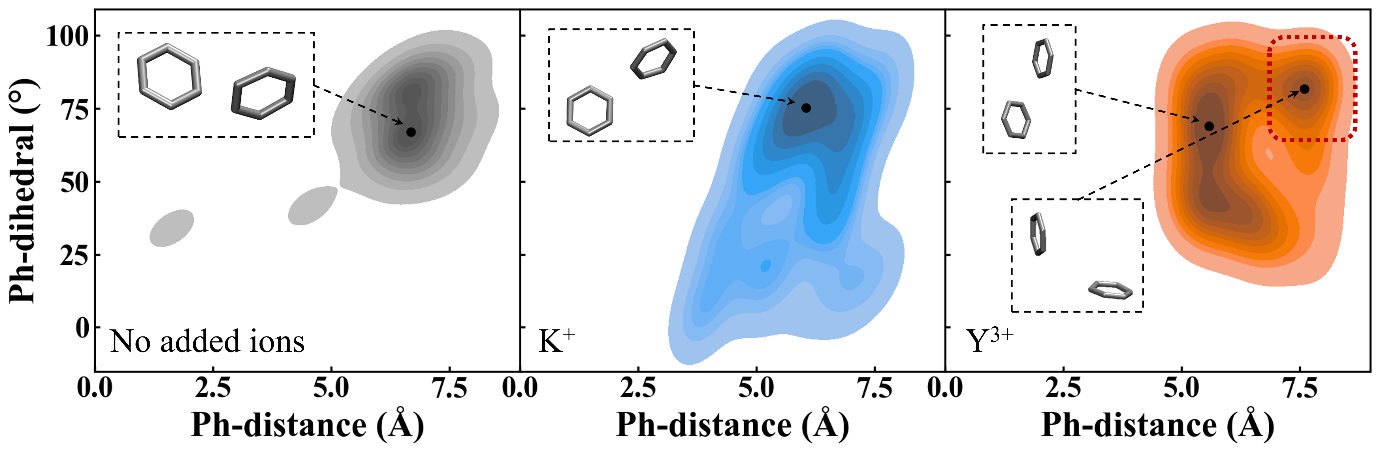


Figure S12 Free energy landscape of phenyl rings distance-dihedral distribution among BB-peptide systems. (Inset) The representative conformation of the phenyl contacts max density point.


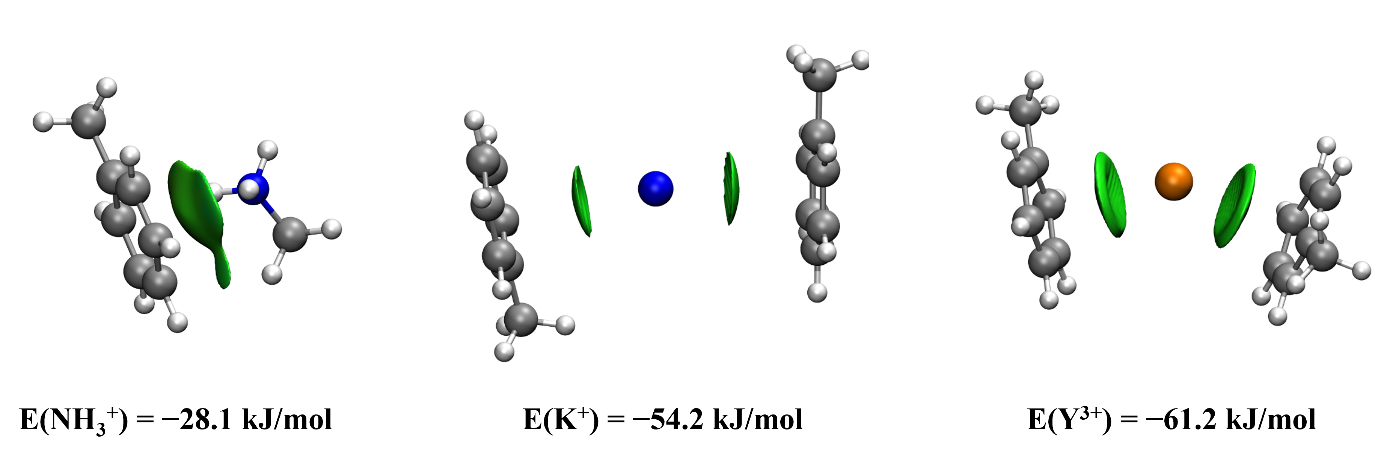


Figure S13 Interaction energy between phenyl rings and cations: NH_3_^+^, K^+^, Y^3+^. The isovalue of independent electron density gradient value was set to 0.0005 a.u. during illustration (while that of NH_3_^+^ was set to 0.005 a.u.). The interaction energy value was calculated between the cations and the toluene part.


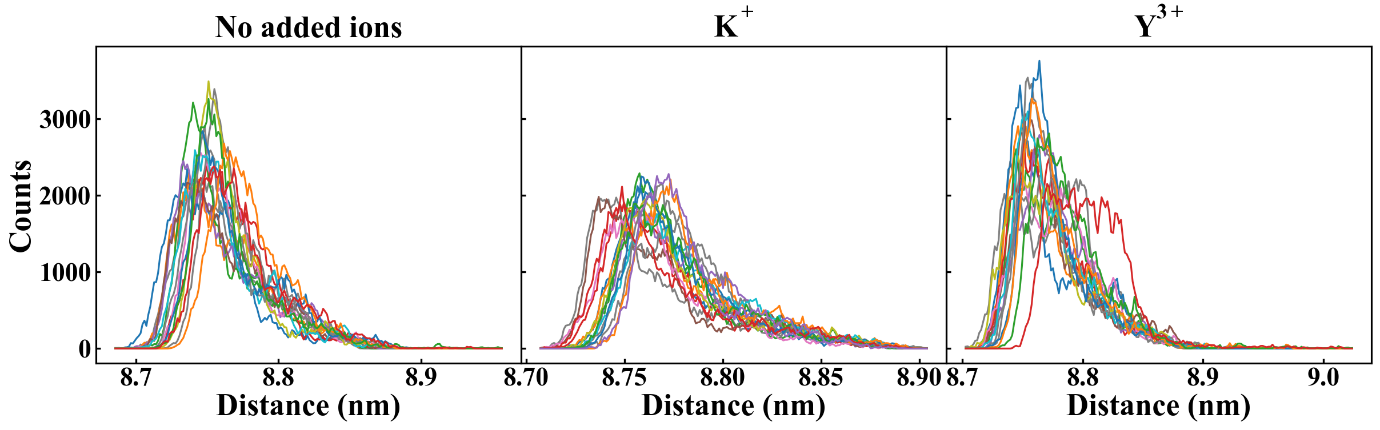


Fig. S14 Histograms of the umbrella sampling simulations: (A) No added ions, (B) K^+^, and (C) Y^3+^.

1] Y. Cong, M. Vatankhah-Varnosfaderani, V. Karimkhani, A. N. Keith, F. A. Leibfarth, M. R. Martinez, K. Matyjaszewski, S. S. Sheiko, *Macromolecules* **2020**, 53, 8324.

[2] C.-S. Wang, D. A. Pham, H. Zhang, J.-M. Rabanel, N. Hassanpour, X. Banquy, *Advanced Functional Materials* **2024**, 34, 2402960.
